# The Phytochrome B/Phytochrome C Heterodimer Is Necessary for Phytochrome C-Mediated Responses in Rice Seedlings

**DOI:** 10.1371/journal.pone.0097264

**Published:** 2014-05-22

**Authors:** Xianzhi Xie, Takatoshi Kagawa, Makoto Takano

**Affiliations:** 1 Photobiology and Photosynthesis Research Unit, National Institute of Agrobiological Sciences, Tsukuba, Ibaraki, Japan; 2 Shandong Rice Research Institute, Shandong Academy of Agricultural Sciences, Jinan, Shandong, China; University of Texas at Austin, United States of America

## Abstract

**Background:**

PhyC levels have been observed to be markedly lower in *phyB* mutants than in *Arabidopsis* or rice wild type etiolated seedlings, but the mechanism of this phenomenon has not been fully elucidated.

**Results:**

In the present study, we investigated the mechanism by which phyB affects the protein concentration and photo-sensing abilities of phyC and demonstrated that rice phyC exists predominantly as phyB/phyC heterodimers in etiolated seedlings. PHYC-GFP protein was detected when expressed in *phyA phyC* mutants, but not in *phyA phyB* mutants, suggesting that phyC requires phyB for its photo-sensing abilities. Interestingly, when a mutant *PHYB* gene that has no chromophore binding site, *PHYB*(*C364A*), was introduced into *phyB* mutants, the phyC level was restored. Moreover, when *PHYB(C364A)* was introduced into *phyA phyB* mutants, the seedlings exhibited de-etiolation under both far-red light (FR) and red light (R) conditions, while the *phyA phyB* mutants were blind to both FR and R. These results are the first direct evidence that phyC is responsible for regulating seedling de-etiolation under both FR and R. These findings also suggest that phyB is indispensable for the expression and function of phyC, which depends on the formation of phyB/phyC heterodimers.

**Significance:**

The present report clearly demonstrates the similarities and differences in the properties of phyC between *Arabidopsis* and rice and will advance our understanding of phytochrome functions in monocots and dicots.

## Introduction

Plants sense diverse light signals from the environment via a family of plant photoreceptors, including phytochromes, cryptochromes, and phototropins. Phytochromes are chromoproteins that regulate the expression of a large number of light-responsive genes and thus influence many photomorphogenic events [1]–[5].

The phytochrome monomer is an approximately 120 kDa protein attached to a linear tetrapyrrole chromophore, and the phytochrome molecule is thought to exist as a dimer in two stable photointerconvertible forms, Pr and Pfr. Photochromicity between inactive, red light (R)-absorbing Pr and active, far-red light (FR)-absorbing Pfr endows phytochromes with the capacity to sense the relative ratio of R and FR [6]. Phytochromes in higher plants are encoded by small gene families [7], [8]. Molecular phylogenetic analyses indicate that the angiosperm phytochrome gene family is composed of four subfamilies, *PHYTOCHROME A* (*PHYA*), *PHYB*, *PHYC/F*, and *PHYE* [9]. In *Arabidopsis*, *PHYD* is further derived from an ancestral *PHYB* gene by a recent gene duplication event [7], and as a result, *Arabidopsis* has five *PHY* genes, *PHYA* to *PHYE* [7], [10].

phyA and phyB exist as homodimers in wild type (WT) and phytochrome-overexpressing lines of *Arabidopsis* [11], [12], and the native complement of phytochromes in plants has often been assumed to consist of only homodimeric forms. However, in *Arabidopsis*, heterodimers of type II phytochromes have been observed *in vivo* [13]. Furthermore, there is no evidence for the homodimerization of phyC or phyE, indicating that these two phytochromes are present in cells only as heterodimers [14]. The formation of such heterodimeric phytochromes increases the potential complexity of R/FR light-sensing and signaling mechanisms in plants.

In rice (*Oryza sativa*), the phytochrome gene family is composed of three members, *PHYA*, *PHYB*, and *PHYC* [15]–[18]. Recently, the generation and characterization of rice phytochrome single, double, and triple mutants have revealed that individual members of the rice phytochrome family have synergistic, as well as overlapping, functions in the control of the responses to R and FR in terms of de-etiolation and flowering processes [19]–[22]. Under continuous FR, *phyA* mutants exhibited partially impaired de-etiolation, and *phyA phyC* double mutants exhibited no significant residual phytochrome responses, indicating that both phyA and phyC are involved in the photo-sensing of FR in rice [20]. However, *phyB phyC* double mutants did not show any apparent decrease in their sensitivity to FR compared with *phyB* mutants, indicating that the mutation of phyC in the phyB-deficient background did not have any additive effect. Moreover, the responses to FR were completely canceled in *phyA phyB* double mutants. It has also been reported that seedlings of *phyA phyB* double mutants are blind to R and that *phyB phyC* and *phyB* mutants showed similar sensitivity to R regarding de-etiolation responses [20]. These observations implied that phyB somehow affects phyC in the photo-sensing of FR or R in rice.

The dependence of phyC on phyB has been reported in *Arabidopsis*. Monte et al. found that phyB is necessary for the function of phyC in mediating the responses to R in *Arabidopsis* and that the functional dependency of phyC on phyB correlates with constitutively lower levels of phyC in *phyB* mutants compared with in WT [23], which was observed in several reports [24], [25]. These observations could result from the reduced stability of phyC when phyB is absent, preventing heterodimer formation [13], [14].

In rice, it has also been reported that phyC levels are lower in *phyB* mutant than WT etiolated seedlings and that phyB affects the photo-sensing ability of phyC [20]. In this study, we demonstrated that phyB and phyC form heterodimers in rice, consistent with observations in *Arabidopsis*. To reveal the properties of phyB/phyC heterodimers in rice, two types of transgenic plants overexpressing phyC-GFP or chromophore-less PHYB were generated and examined for their responsiveness to light. Our results revealed that phyB is indispensable for phyC stability and for its ability to sense both R and FR in the control of de-etiolation in rice seedlings. This study provides a new molecular mechanism for the interaction among phytochromes in the photoregulation of diverse developmental processes.

## Materials and Methods

### Plant Materials

Plant materials used in this study were as follows: WT, *Oryza sativa* L. cv. Nipponbare; *phyA* mutants, *phyA-4*; *phyB* mutants, *phyB-1*; *phyC* mutants, *phyC-1*; *phyA phyC* mutants, *phyA-4 phyC-1*; and *phyA phyB* mutants, *phyA-4 phyB-1* [19], [20]. The background of these mutants is Nipponbare. *Arabidopsis* is in the gl-1 genetic background.

### Protein Expression in *Escherichia coli* and Protein Purification

For quantifying the relative levels of phyB and phyC proteins, phyB-His and phyC-His were expressed in *Escherichia coli* as described by Takano et al. [20]. Dilutions of purified proteins were separated by SDS-PAGE and stained with Coomassie Brilliant Blue [26]. Signal intensities of purified proteins were analyzed using NIH image 1.62.

To facilitate phyA or phyC apoprotein binding to chromophores in *E. coli*, the protein was expressed with a calmodulin binding domain at its N-terminus and a 6-histidine tag at its C-terminus in phytochromobilin-expressing Rosetta 2 cells (Novagen, Merck KGaA, Darmstadt, Germany) and purified, as previously described [27].

### Immunoblotting and Co-immunoprecipitation

Soluble protein was extracted from above-ground parts of etiolated or light-grown seedlings as described by Takano et al. [19]. Protein extracts were separated on SDS-PAGE and blotted onto PVDF membranes (Immobilon-P, Millipore, MA). phyA, phyB, and phyC proteins were detected immunochemically using a colorimetric detection method (NBT/BCIP stock solution, Roche Applied Science, Basel, Switzerland) or ECL chemiluminescence kits (GE Healthcare, Uppsala, Sweden). For immunoblot analysis of *Arabidopsis* phyA protein, monoclonal antibody, AA01, was used.

For co-immunoprecipitation (co-IP), 200 µg of protein extract from dark-grown seedlings or 500 µg from continuous white light (cW)-grown seedlings were precleared by adding 50 µL of γProteinA-Sepharose Fast Flow (GE Healthcare), incubating for 30 min at 4°C, and centrifuging at 15,000 rpm for 10 min. The γProteinA-Sepharose-anti-PHYC conjugates were prepared by mixing 20 µL of γProteinA-Sepharose with anti-PHYC antibody or preimmune serum from the same rabbit, gently rocking for 1 h at 4°C, and washing twice with PBS buffer (137 mM NaCl, 2.7 mM KCl, 10 mM Na_2_HPO_4_, 2 mM KH_2_PO_4_). Then, precleared protein extracts were added to the Sepharose-anti-PHYC conjugates and incubated for 3–4 h at 4°C. The Sepharose-anti-PHYC-protein complex was washed six times with NETN buffer (100 mM NaCl, 1 mM EDTA, 20 mM Tris-Cl pH 8.0, 0.5% (v/v) Nonidet P-40) containing 900 mM NaCl and twice with NETN buffer without additional NaCl. Proteins bound to the Sepharose beads were eluted by boiling for 5 min in 2× SDS sample buffer. The eluted proteins were analyzed by immunoblotting as described in Takano et al. [19]. Immunochemical detection was performed using an immunoblot kit (alkaline phosphatase system, using 5-bromo-4-chloro-3-indolyl phosphate/nitro blue tetrazolium (BCIP/NBT) stock solution; Roche Applied Science).

### Size Exclusion Chromatography

Protein extracts from WT, *phyB*, and *phyA phyC* rice seedlings or WT *Arabidopsis* seedlings grown in the dark for 8 days were prepared, and 0.25-mL samples containing 1 mg of total soluble protein were applied to a Superdex 200 HR 10/30 (GE Healthcare). The column was eluted with buffer (50 mM Na-phosphate, pH 7.0, 150 mM NaCl) at 4°C at a rate of 0.25 mL/min, and 0.5 mL fractions were collected. Co-IP and immunoblot analyses of each fraction were performed as described by Takano et al. [19]. The column was calibrated with protein molecular weight standards (GE Healthcare). The elution volume-molecular weight curve was plotted. The plotted data were fitted with a regression line, and the molecular weight of each fraction was estimated based on the linear regression line.

### Plasmid Construction and Rice Transformation

For the construction of the mutant *PHYB(C364A)*, the codon (TGC) encoding the chromophore-binding site (cysteine) was substituted with GCC encoding alanine by site-directed PCR. Rice *PHYB* full-length cDNA or mutated *PHYB(C364A)*cDNA was subcloned into the pPZP2Ha3 vector [28] in the sense orientation. These plasmids were introduced into *Agrobacterium tumefaciens* strain EHA101 by electroporation.

To construct the *PHYC-GFP* (*PCG*) overexpressing lines, rice *PHYC* cDNA was amplified by PCR with a nucleotide substitution that replaced the *PHYC* translation termination codon (TAG) with an oligonucleotide sequence containing a KpnI site. The *PHYB* moiety of *Arabidopsis* phyB-GFP fusion construct [29] was replaced with *PHYC* to obtain the *PCG* vector, in which the *PHYC-GFP* fusion sequence was inserted between the constitutive cauliflower mosaic virus 35S promoter and the Nos terminator of pPZP211/35S-nosT26 [30]. This plasmid was introduced into *Agrobacterium tumefaciens* strain EHA105 [31] by electroporation.

Rice (*Oryza sativa* L. Cv. Nipponbare) was transformed via the agroinfection methods of Hiei et al. [32]. The *PHYA* allele is heterozygous in the host *phyB* mutants, permitting the creation of *phyA phyB* double mutants in the progeny of the overexpressor.

### Determination of Mutant Alleles in the Transgenic Lines

DNA was extracted from seedlings of transgenic lines and used as templates for PCR. For the *phyA-4* lines, the primer pair phyA BF (5′ -CCAACATCATGGACCTTGTG-3′)/phyA HR (5′ -CCATTGACCAATCCATTGCT-3′) was used to detect the WT allele, and phyA BF/T17 R1 (5′ -CAGCAACGATGTAGATGGTCAAGC-3′) was used to detect the mutant allele (insertion of *Tos17*). To detect insertion of *PHYB(C364A)* and *PHYB* in transgenic lines, the primer pair phyB Fw2032 (5′ -GAGACAGCAACAGTACCCATCTTTG- 3′)/phyB R1 (5′-CTTCCCCTCTTGACCATCCT-3′) was used to amplify an approximately 600-bp fragment of *PHYB*. To detect insertion of *PHYC-GFP*, the primer pair 35S Fw (5′ -TGACGTAAGGGATGACGCACAATC-3′)/phyC Rv444 (5′ -GGGGTTGAGCAGGTTGACG-3′) was used. PCR reagents were obtained from the Qiagen Taq system (QIAGEN GmbH, Germany).

### RNA Analysis

For analyzing light-induced gene expression, total RNAs were extracted from the seedlings using an RNeasy Plant Mini Kit (QIAGEN, GmbH). Expression of *ribrose-1,5-bisphosphate carboxylase small subunit* (*RbcS*) was analyzed by RNA blot analysis as described by Takano et al. [20]. RT-PCR was used to examine the expression levels of two *light-harvesting chlorophyll a/b binding protein* (*Lhcb*) genes, *Os03g0592500* and *Os09g0346500*. For *Os03g0592500*, the primer pair used was F1 (5′ -TGAGCACAAC GACACGAT-3′)/R1 (5′ -TCTCCTCGATCGATCACA-3′). For *Os09g0346500*, the primer pair used was F1 (5′ -GTAGCTTAGCAGTGGTTAATTGT-3′)/R1 (5′ -TCTTCATCTTCTTAGTfGTACACAAC-3′). The rice ubiquitin gene (*UBQ*) was used as an internal control, and its primer pair was 237F (5′ -GAGCCTCTGTTCGTCAAGTA-3′)/304R (5′ -ACTCGATGGTCCATTAAACC-3′). The PCR conditions were 94°C for 3 min and 20 cycles of 94°C for 1 min, 50°C, 45°C, or 55°C for 1 min for *Os03g0592500*, *Os09g0346500*, or *UBQ*, respectively, and 72°C for 1 min. PCR reagents were obtained from the Qiagen Taq system.

For analyzing gene expression in *PCG*/*aabb* lines, *PCG*/*Aabb* transgenic lines with homozygous *PHYC-GFP* were harvested and rapidly frozen in liquid nitrogen. Then, the individual seedlings were numbered. A small portion from each seedling was used for analyzing the genotype to distinguish the *phyA-4* mutant allele from the WT allele by PCR, as described above. The left portion of *PCG*/*aabb* seedlings were used for RNA analysis.

### Spectrophotometric Assays

Nine-day-old dark grown seedlings of rice were harvested and frozen with liquid nitrogen. The frozen samples were powdered by a mortar and pestle and suspended in 10 volumes of extraction buffer (100 mM Tris-HCl pH 7.5, 5 mM EDTA pH 8.0, 0.2% 2-mercaptoethanol, and protease inhibitor cocktail (Complete, Roche Diagnostics GmbH, Mannheim, Germany)). After being filtered with two layers of Miracloth (Calbiochem, Merck KGaA, Darmstadt, Germany), the suspension was centrifuged for 10 min at 15,000 *g* and 4°C. The supernatant was combined with solid ammonium sulfate (230 g L^−1^) to concentrate the phytochrome [33] and centrifuged for 30 min at 15,000 *g* after incubation on ice for at least 30 min. The precipitate was re-suspended with re-suspension buffer (100 mM Tris-HCl pH 7.5, 5 mM EDTA). The re-suspension was clarified with centrifugation for 10 min at 15,000 *g*, and the supernatant was used for the measurement.

Spectrophotometric measurements of phytochrome were performed with a photometer (Biospec 1650, Shimazu, Japan). Red and far-red illumination was obtained using red (660 nm; SLP-838A-37, Sanyo Semiconductor Corp., Japan) and far-red (771 nm; HE7601SG, Hitachi, Japan) light-emitting diodes.

### Measurement of Coleoptile Length

Sterilized seeds of WT, mutant, or transgenic lines were sown in 0.6% (w/v) agar and then grown in the dark, under FR, R, or white light (W) at 28°C for eight days. Images of the seedlings were captured using a scanner (EPSON GP9600, SEIKO EPSON, Suwa, Japan), and coleoptile lengths were measured by scale.

For analyzing the coleoptile length of *PCG*/*aabb* lines, we sowed the seeds of PCG/Aabb transgenic lines. After seedlings had grown for eight days, we labeled and measured the coleoptile length of individual seedlings. Then, we analyzed the genotype of individual seedlings to distinguish the *phyA-4* mutant allele from the WT allele by PCR, as described above.

### Growth Conditions for the Flowering Time Measurements

Nipponbare, *phyB*, and *PHYB(C364A)*/*Aabb* lines were grown in a growth chamber in LD conditions (light cycle, 14.5 h of light/9.5 h of dark; 28°C in the day/23°C at night). The light sources were metal halide lamps (MLBOC400C-U, MITSUBISHI/OSRAM; 390 µmol m^−2^ s^−1^).

### Treatment with MG132

The stock solution of 10 mM MG132 was prepared by dissolving MG132 in dimethyl sulfoxide (DMSO; Sigma-Aldrich, MO). Four-day-old dark-grown seedlings were pretreated with either 0.5% DMSO or 50 µM MG132 (Calbiochem). After 1.5 h of pretreatment, seedlings were transferred to R light for 6 h. The soluble protein was extracted from seedlings and analyzed by western blotting using anti-PHYA or anti-PHYC antibodies [20].

### Light Sources and Light Intensities

Monochromatic light sources and W in this study were the same as those described by Takano et al. [20]. The fluence rates were 15 µmol m^−2^ s^−1^ for FR, 15 µmol m^−2^ s^−1^ for R, and 50 µmol m^−2^ s^−1^ for W.

Sequence data from this article can be found in the GenBank/EMBL data libraries under accession numbers AB109892 (*PHYB*), AB018442 (*PHYC*), X07515 (*RbcS*), and AY072820 (*UBQ*).

## Results

### Quantification of the Relative Levels of phyB and phyC Proteins in Rice Seedlings

To analyze the interaction between phyB and phyC, we initially quantified the relative levels of phyB and phyC in WT rice seedlings grown under either continuous darkness or cW by comparative western blotting by using purified proteins as standards (Fig. S1).

Protein extracts from 5-day-old etiolated seedlings and dilution series of standard proteins (PHYB-His or PHYC-His) were separated by SDS-PAGE on the same gel, and phyB and phyC were detected by anti-PHYB and anti-PHYC antibodies, respectively. For each blot, signal intensities of phyB or phyC for the same protein extracts were compared with those of standard proteins (Figs. S1B and S1C). The phyB to phyC ratio was estimated to be 1.3∶1 in the etiolated seedlings (for calculation details, see supporting information online).

The same methods were applied to quantify the relative level of phyB:phyC in 7-day-old seedlings grown under W. However, the immunoblot signals of phyC protein were too low to estimate the phyC level because rice phyC is light-labile. The protein concentration of phyC was reduced to about one-sixteenth of the level in etiolated seedlings after 24-h growth under W, whereas phyB was light-stable, only less than a one-fourth reduction of levels in etiolated seedlings after growth for 24 h under W (Fig. S2).

### phyB and phyC Form Heterodimers in Rice Seedlings

**Figure 1:**
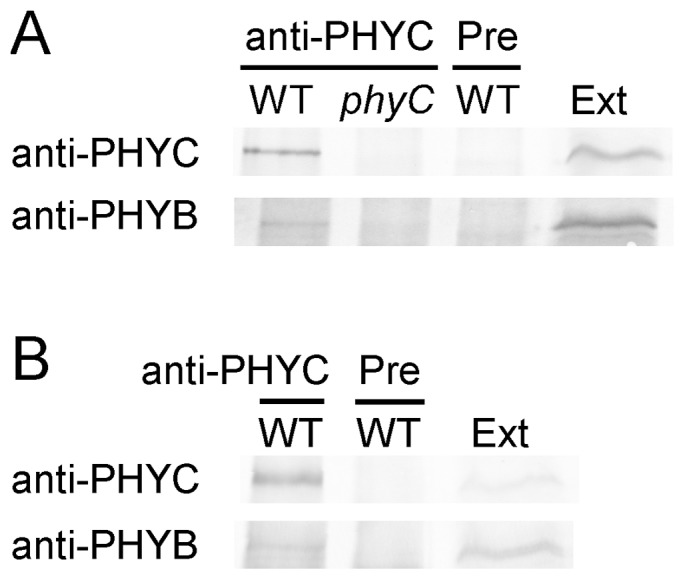
phyB and phyC proteins form complexes in both etiolated and light-grown rice seedlings. **A.** Protein extracts (200 µg) from 7-day-old etiolated seedlings of WT or *phyC* mutants (*phyC*) were immunoprecipitated with an anti-PHYC antibody (Anti-PHYC/WT; Anti-PHYC/*phyC*) or with preimmune serum from the same rabbit (Pre/WT). phyC or phyB was immunodetected in the precipitation. Thirty micrograms of protein extracts from WT were loaded as the positive control (Ext). **B.** Protein extracts (500 µg) from WT seedlings grown under W for 7 days were immunoprecipitated with an anti-PHYC antibody (Anti-PHYC/WT) or with preimmune serum from the same rabbit (Pre/WT). phyC or phyB was immunodetected in the precipitation. Fifty micrograms of protein extracts from WT were loaded as the positive control (Ext).

It has been reported that phyB and phyC form heterodimers in *Arabidopsis* [13], [14]. To examine the physical interaction between phyB and phyC in rice, co-IP experiments were performed using protein extracts from rice seedlings grown under either dark or W conditions (Fig. 1). When protein extracts from WT etiolated seedlings were used, phyB was co-immunoprecipitated with anti-PHYC antibody (Fig. 1A, anti-PHYC/WT). No phyB was co-immunoprecipitated in the control experiments using protein extracts from *phyC* seedlings (Fig. 1A, anti-PHYC/*phyC*) or preimmune serum as the precipitating antibody (Fig. 1A, Pre/WT). The interaction between phyC and phyB was also detected in W-grown WT seedlings (Fig. 1B), although the co-IP signals were weaker than those of etiolated seedlings. These results indicate that a potential physical interaction exists between phyB and phyC in both etiolated and light-grown rice seedlings.

However, the question remains whether phyB and phyC form heterodimers in rice seedlings as observed in *Arabidopsis* [13], [14]. To address this question, protein extracts from etiolated seedlings of WT or phytochrome mutants were fractionated by SEC. The column was calibrated with protein molecular weight standards, and the molecular size of each fraction was calculated based on the calibration line.

**Figure 2:**
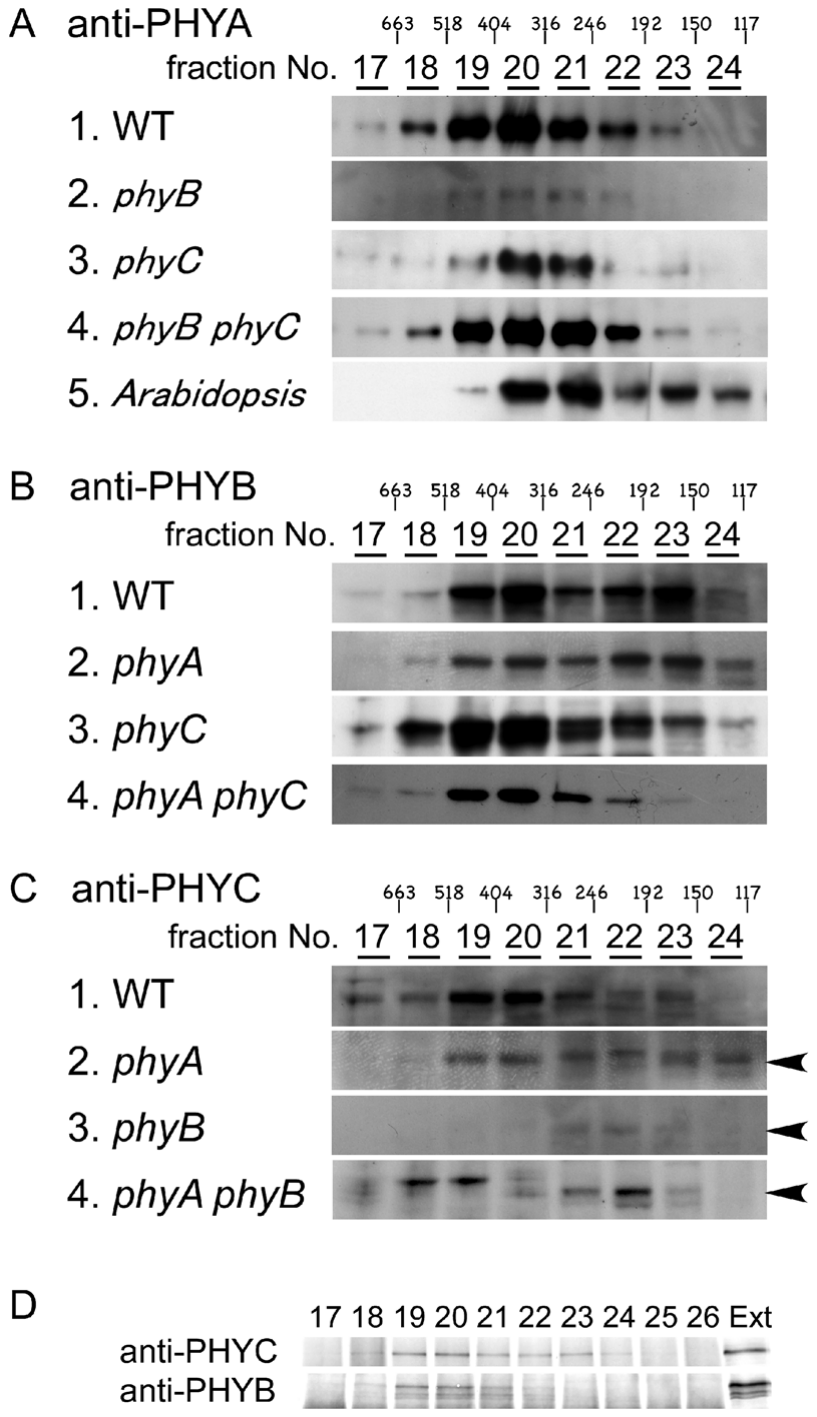
Immunoblot analyses of phyA, phyB, and phyC in the column fractions of protein extracts. Protein was extracted from 7-day-old etiolated seedlings of WT and all phytochrome single and double mutants and fractionated by SEC. phyA, phyB, and phyC were immunochemically detected with anti-PHYA (**A**), anti-PHYB (**B**), and anti-PHYC antibodies (**C**), respectively, in the individual fractions (#17–#24). Small numbers above the fraction numbers are the molecular sizes which were calculated based on the calibration line of standard proteins. *Arabidopsis* seedlings were used to characterize the migration of homodimer phyA. **D.** Physical interactions between phyB and phyC in the fractions (#17–#26) of protein extracts from 7-day-old etiolated seedlings of WT. The individual fractions were immunoprecipitated with an anti-PHYC antibody. phyB and phyC in the precipitates were detected by immunoblotting. Thirty micrograms of protein extracts from WT were loaded as the positive control (Ext).

**Figure 3:**
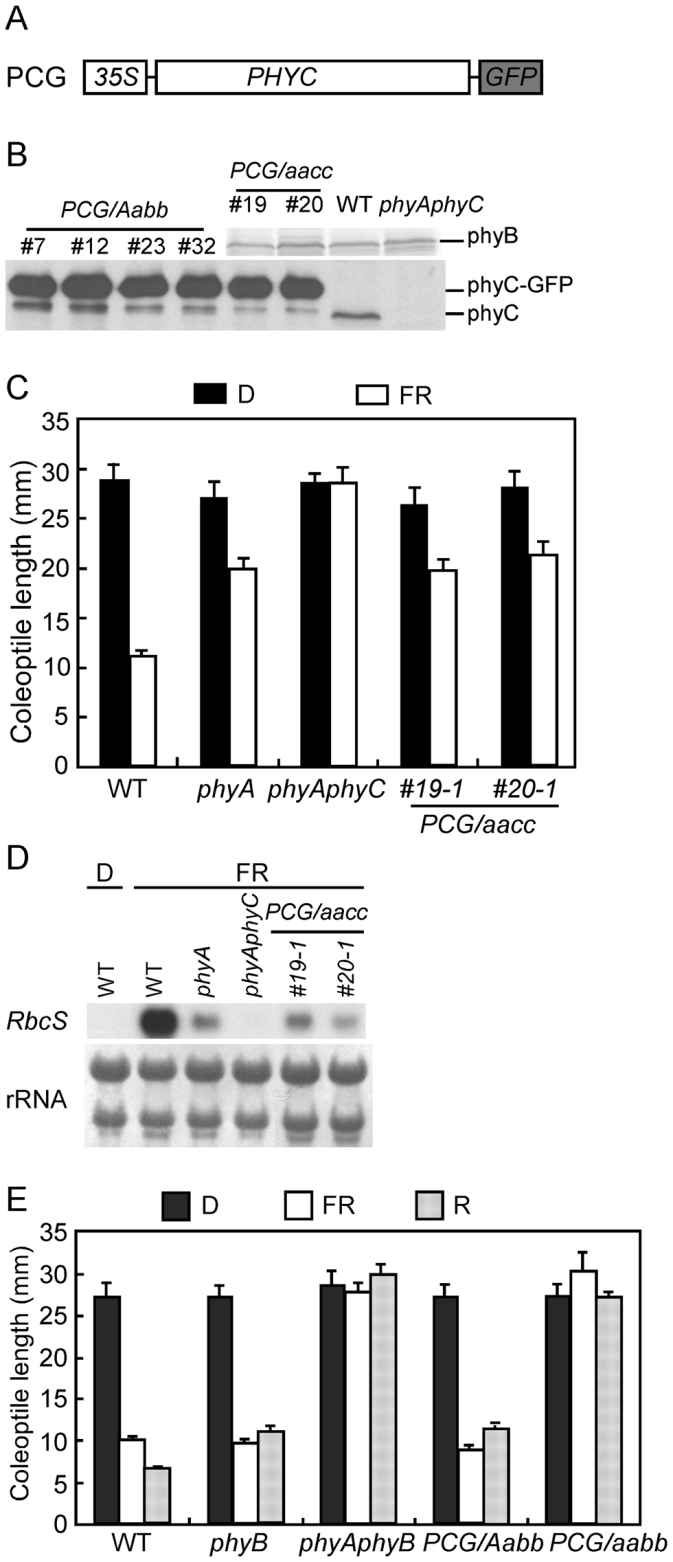
phyC-GFP is biologically active in *phyA phyC* backgrounds but inactive in phyB-mutant backgrounds. **A.** Diagram of the *PHYC-GFP* fusion construct introduced into *phyA phyC* and *phyB* mutants. **B.** Immunoblot analyses of phyB and the phyC-GFP fusion protein in WT, *phyA phyC*, and *PCG*/*aacc* transgenic lines using anti-PHYB and anti-PHYC antibodies. Protein extracts (25 µg from *PCG/aacc* lines and *PCG/Aabb* lines; 50 µg from WT and *phyA phyC* seedlings) were loaded to detect phyC-GFP and phyC. Fifty micrograms of protein were loaded to detect phyB. *Aabb* is the *phyB* mutant where the *PHYA* mutant allele is heterozygous. **C.** The phyC-GFP fusion protein caused inhibition of coleoptile growth by FR irradiation in *PCG/aacc* transgenic lines. Mean coleoptile lengths are shown for WT, *phyA*, *phyA phyC*, and *PCG/aacc* seedlings (#19 and #20) grown under FR (15 µmol m^−2^ s^−1^, open bars) or in the dark (filled bars) for 8 days. The mean ± SE (standard error) obtained from at least 12 seedlings is plotted. **D.** phyC-GFP fusion protein exerted the phyC function in the expression of *RbcS*. The expression of *RbcS* induced by FR was comparatively analyzed by RNA blotting in the seedlings of WT, *phyA*, *phyA phyC*, and *PCG/aacc* (#19 and #20). *RbcS* was used as a probe. rRNA was stained by methylene blue as a quantity control. **E.** phyC-GFP did not participate in the photoinhibitory responses of coleoptile growth under FR or R in the *phyB* mutant background. Mean coleoptile lengths are shown for WT, *phyB*, *phyA phyB*, *PCG*/*Aabb*, and *PCG*/*aabb* seedlings grown under FR (15 µmol m^−2^ s^−1^, open bars) or under R (15 µmol m^−2^ s^−1^, hatched bars) or in the dark (filled bars). The mean ± SE obtained from at least 12 seedlings is plotted, excluding the *PCG/aabb* genotype, for which only six seedlings were grown.

**Figure 4:**
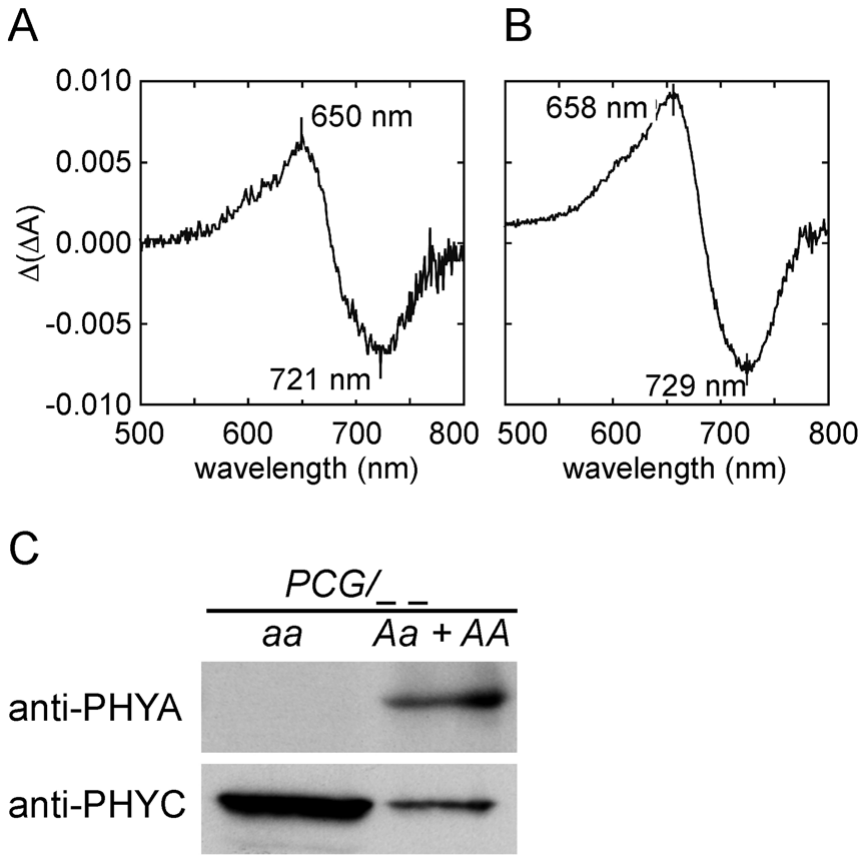
Absorption difference spectra of phyC-GFP. Difference spectrum characteristics of phytochromes in protein extracts from 7-day-old etiolated seedlings of *PCG/aabb* (**A**) or *PCG/Aabb* (**B**). Difference spectra were measured using a double-beam spectrophotometer and normalized by 1 mg total protein per mL. **C.** Immunoblot analyses of phyA and the phyC-GFP fusion protein in *PCG/Aabb* or *PCG/aabb* transgenic lines using anti-PHYA and anti-PHYC antibodies. Each lane was loaded with 5 µg of total protein.

**Figure 5:**
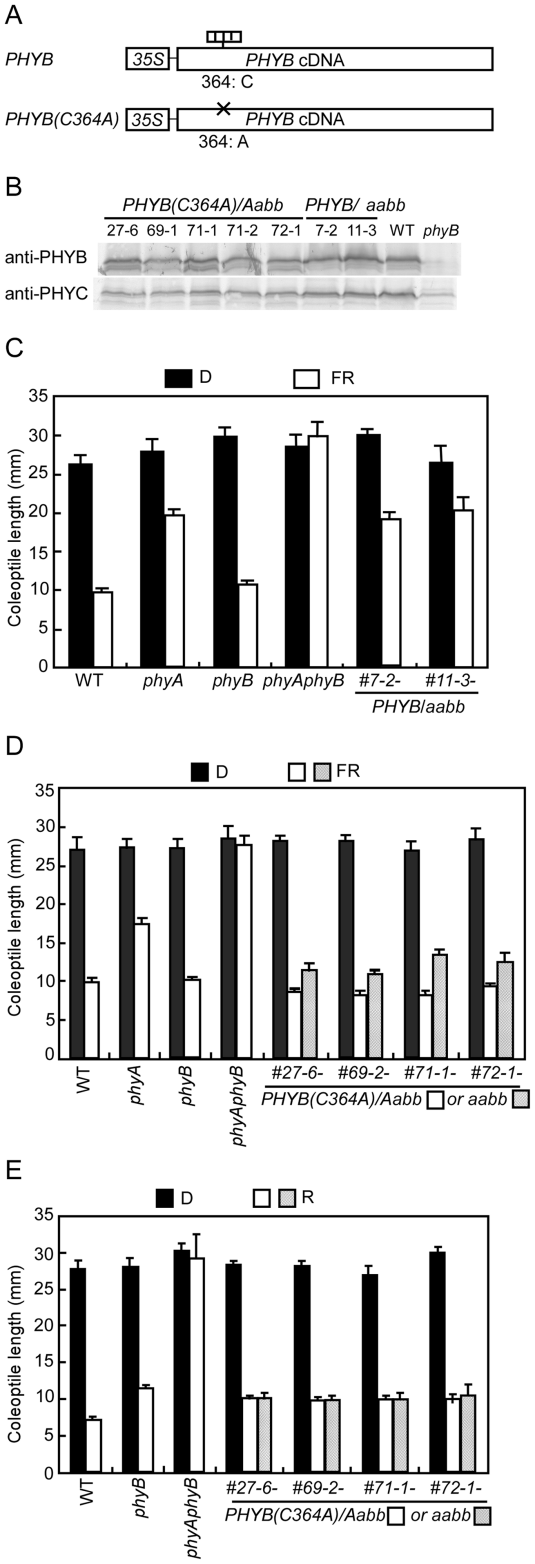
phyC is biologically active in *PHYB/aabb and PHYB(C364A)/aabb* transgenic lines. **A.** Diagram of *PHYB* and mutant PHYB(*C364A*) constructs introduced into *phyB* mutants. **B.** Immunoblot analyses of phyB and phyC levels in the dark-grown seedlings of *PHYB*/*Aabb* and *PHYB(C364A)*/*Aabb* transgenic lines. Protein extracts from 7-day-old etiolated seedlings of different transgenic lines of *PHYB*(*C364A*)/*Aabb* (#27, #69, #71, and #72) and *PHYB*/*aabb* (#7 and #11) were probed with anti-PHYC and anti-PHYB antibodies. WT and *phyB* were used as positive and negative controls, respectively. Each lane was loaded with 50 µg of protein extracts. **C.** FR inhibited coleoptile growth in *PHYB*/*aabb* transgenic lines. Mean coleoptile lengths were shown for the seedlings of WT, *phyA*, *phyB*, *phyA phyB*, and two lines of *PHYB*/*aabb* (#7-2- and #11-3-) grown under FR (15 µmol m^−2^ s^−1^, open bars) or in the dark (filled bars) for eight days. The mean ± SE obtained from at least 12 seedlings is plotted. **D** and **E.** FR (**D**) or R (**E**) inhibited coleoptile growth in the *PHYB(C364A)/Aabb* and *PHYB(C364A)/aabb* transgenic lines. Mean coleoptile lengths are shown for WT, *phyA* (only in **D**), *phyB*, *phyA phyB*, *PHYB(C364A)/Aabb*, and *PHYB(C364A)/aabb* seedlings grown under FR (15 µmol m^−2^ s^−1^, **D**), R (15 µmol m^−2^ s^−1^, **E**), or in the dark (filled bars) for eight days. Four independent lines (#27-6-, #69-2-, #71-1-, and #72-1-) of *PHYB(C364A)* were used, and results from different genetic backgrounds (*Aabb* or *aabb*) were depicted with separate bars. The mean ± SE obtained from at least 12 seedlings is plotted.

Using the same column with the same conditions, protein extracts from etiolated seedlings of WT rice and various phytochrome mutants were fractionated. Aliquots of each eluted fraction were separated by SDS-PAGE, and phyA, phyB, and phyC were immunochemically detected. As shown in Figures 2A-1, 2B-1, and 2C-1, phyA, phyB, and phyC from WT rice were mainly detected in Fraction #20 with a calculated molecular mass in the range of 316 to 404 kDa, similar to observations in *Arabidopsis* where all five native phytochrome proteins migrate at apparent masses in the range of 300 to 380 kDa on SEC [13]. We characterized the migration of phyA from etiolated *Arabidopsis* seedlings using the same assay conditions, and confirmed that *Arabidopsis* phyA was mainly detected in Fractions #20 and #21 (Fig. 2A-5). Since *Arabidopsis* phyA is a homodimer [13], we concluded that rice phyA, phyB, and phyC predominantly exist in dimeric forms. In WT and *phyA*-mutant extracts, phyB was detected in the monomer fractions as a second peak (Fig. 2B-1 and 2B-2). In phyC-deficient mutants, phyB was only detected in dimeric forms (Fig. 2B-3 and 2B-4), suggesting that the absence of phyC prevents monomerization of phyB. As dimerization is necessary for full activity of phyB [12], [34], it is hard to imagine that bioactive phyB exists as monomers within cells. It remains possible that phyB/phyC dimers are not as stable as phyB homodimers and a small proportion of heterodimers may dissociate during size exclusion chromatography (SEC). To dissect the dimer composition of phyC in WT rice, protein extracts from etiolated seedlings of various phytochrome mutants were fractionated by SEC and phyC was detected. In the *phyA* mutant, phyC migrated in a position equivalent to a dimer, the same as in WT (Fig. 2C-2). However, in the phyB-deficient mutants (*phyB* and *phyA phyB* mutants), phyC was mainly detected as a putative monomer in Fraction #22 with a molecular mass of 192 to 246 kDa (Figs. 2C-3 and 2C-4). Thus, phyB is necessary for forming a phyB/phyC heterodimer and phyC exists in a monomeric form in the absence of phyB.

The physical interaction between phyB and phyC was also supported by co-IP assays in the eluted fractions. When precipitated with an anti-PHYC antibody, phyC was detected in Fractions #18 to #22 (Fig. 2D, anti-PHYC), and phyB was co-immunoprecipitated in the fractions corresponding to the dimeric complexes (Fig. 2D, anti-PHYB). These results further confirm that phyB and phyC form heterodimers, like those observed in *Arabidopsis* [14].

These deductions were supported by *in vitro* experiments using recombinant proteins. Recombinant phyC with phytochromobilins as a chromophore was expressed in *E. coli* (Fig. S3A). As a control, recombinant phyA was also expressed using the same experimental procedure. The recombinant phyA and phyC showed typical R/FR difference spectra with maximum peaks of 667 nm and 650 nm, and minimum peaks of 724 nm and 725 nm, respectively (Figs. S3B and S3C), indicating that recombinant phyA and phyC are spectrophotometrically active. When recombinant phyA or phyC was fractionated by SEC using the same column, recombinant phyA was mainly detected in the dimeric Fraction #20, whereas recombinant phyC was mainly detected in the monomeric Fraction #24 (Fig. S3D) as detected using both anti-His and phytochrome-specific antibodies. These results suggest that rice phyC exists as a monomer, while phyA exists as a homodimer.

### phyC-GFP is biologically active in *phyA phyC* mutants, but inactive in *phyB* mutants

Studies on phytochrome single and double mutants have revealed that phyC is involved in FR photo-sensing for de-etiolation (e.g. inhibition of coleoptile growth) as well as induction of light-regulated genes, such as *Lhcb* or *RbcS*, in rice [20]. However, in the mutants deficient in phyB, these responses mediated by phyC were negligible, as indicated by the findings that *phyB* and *phyB phyC* showed similar responses to both R and FR and that the *phyA phyB* and *phyA phyB phyC* mutants always exhibited the same phenotype under either FR or R [20], [21]. These observations suggest that phyC function depends on the presence of phyB. phyC protein concentration is greatly reduced in *phyB* mutants [20], so it is possible that reduced phyC levels are responsible for the functional inactivity of phyC in *phyB* mutants. An alternate possibility is that phyC functions only in the phyB/phyC heterodimeric form. To test the first possibility, we produced transgenic plants overexpressing *PHYC-GFP* and examined their responses to light.

To increase the phyC levels in *phyB* mutants, *35S:PHYC-GFP* (Fig. 3A) was introduced into *phyB* mutants, where the *PHYA* allele is heterozygous in the host *phyB* mutant line (*PCG/Aabb*) to obtain progenies of *PHYC* overexpressors in *phyA phyB* double mutants (*PCG/aabb*). The same construct was also introduced into the *phyA phyC* double mutant as a positive control (*PCG/aacc*). Accumulation of phyC-GFP in transgenic seedlings was examined by immunoblot analysis. Protein extracts from the 7-day-old etiolated T2 seedlings of *PCG/Aabb* lines (#7, #12, #23, and #32) and *PCG/aacc* lines (#19 and #20) were probed with anti-PHYB and anti-PHYC antibodies (Fig. 3B). Strong signals corresponding to the phyC-GFP fusion protein were detected around 140 kDa in the transgenic lines (Fig. 3B). Signal intensities of the phyC-GFP in the mutants were significantly higher than those of the intrinsic phyC in WT seedlings. On the other hand, the abundance of phyB protein in the transgenic seedlings were comparable to that of WT (Fig. 3B). These results indicate that phyC-GFP overexpression did not increase phyB levels in the *PCG/aacc* transgenic lines.

First, T2 seedlings of *PCG/aacc* transgenic lines were grown under FR for 8 days to examine the biological activity of the phyC-GFP fusion protein in the *phyA phyC* double mutant background. The *phyA phyC* double mutant did not show any responses to FR (Fig. 3C; Fig. S4A) as reported by Takano et al. [20]. In *PCG/aacc* transgenic seedlings (#19 and #20), however, coleoptile growth was inhibited (Fig. 3C; Fig. S4A), and *RbcS* or *Lhcb* was induced by FR to levels similar to those observed in *phyA* mutants (Fig. 3D; Fig. S4B). These results indicate that overexpression of *PHYC-GFP* complemented the loss of phyC function in inhibiting coleoptile growth and inducing light-regulated genes under FR. Therefore, we can conclude that phyC-GFP is biologically active in the *phyA phyC* seedlings.

Second, the biological activities of phyC-GFP were examined in the *phyA phyB* mutant background. Coleoptile growth and induction of *Lhcb* genes were analyzed in the progeny of *PCG/Aabb* plants grown under FR or R for 8 days. Although phyC-GFP fusion protein concentrations in several *PCG/Aabb* transgenic lines (#7, #12, #23, and #32) were as high as those in *PCG/aacc* lines (#19 and #20) in etiolated seedlings (Fig. 3B), the coleoptile lengths of the segregated *PCG/aabb* seedlings grown under either FR or R were the same as the lengths of *phyA phyB* seedlings (Fig. 3E; Figs. S4C and S4E). In addition, coleoptile lengths of the segregated *PCG/Aabb* seedlings were not significantly different from those of *phyB* seedlings under FR or R (Fig. 3E; Figs. S4C and S4E). Furthermore, two *Lhcb* genes, *Os03g0592500* and *Os09g0346500*, were not induced by FR or R in the segregated *PCG/aabb* transgenic seedlings (Figs. S4D and S4F), although phyC-GFP levels in these two lines were comparable with those in *PCG/aacc* seedlings (Fig. 3B). These results indicate that phyC-GFP participated neither in the photoinhibitory responses of coleoptile growth nor in the induction of *Lhcb* genes under FR or R in the phyB-deficient backgrounds (*phyB* or *phyA phyB*). Therefore, the phyC-GFP fusion protein is biologically inactive in the *phyA phyB* and *phyB* transgenic lines. Taken together with the findings that phyC-GFP fusion proteins are biologically active in the *phyA phyC* double mutant (Figs. 3B and 3C), phyB protein is indispensable for the phyC-mediated responses to FR and R. These findings also suggest that reduced phyC levels are not the major factor in its functional loss in phyB-deficient mutants.

The lack of biological responses to FR and R in *PCG/aabb* seedlings might be due to the photochemical inability of expressed phyC-GFP. To examine this possibility, an absorption difference spectrum was obtained. As shown in Figure 4A, a difference spectrum characteristic for the phytochrome was obtained for the protein extract from 7-day-old etiolated seedlings of *PCG/aabb*. The absorption difference spectrum (Fig. 4A) is believed to be that of phyC-GFP because western blotting confirmed that phyC-GFP was the only phytochrome present in *PCG/aabb* seedlings (Fig. 4C). As a reference, a difference spectrum was also obtained from protein extracts of *PCG/Aabb* seedlings (Fig. 4B), where both phyA and phyC are expressed (Fig. 4C). It was also noted by comparing the results of Figures 4A and 4B that maximum and minimum peaks were blue-shifted by 8 nm in phyC-GFP-expressing seedlings compared with those in seedlings expressing a mixture of phyA and phyC-GFP. Such a blue-shift in the difference spectrum of phyC was also observed in our experiments using recombinant phyA and phyC (Fig. S3C), and has been reported in *Arabidopsis* as well [35]. Therefore, phyC-GFP in *PCG/aabb* seedlings is spectrophotometrically active but biologically inactive. These results suggest that phyC can absorb light but not initiate downstream signaling without the presence of phyB. Therefore, we examined the mechanism by which phyB is involved in phyC function.

Because phyB and phyC form heterodimers in the etiolated seedlings, it is assumed that the function of phyC-GFP in *PCG/aacc* seedlings is probably attributed to the formation of phyB/phyC-GFP complexes. Co-IP assays revealed the physical interaction between phyC-GFP and phyB (Fig. S5A). Even so, the mechanism by which phyB affects phyC-mediated responses in the phyB/phyC heterodimer remains elusive. It is unclear whether the binding of phyB to phyC alone is sufficient or whether photo-sensing by phyB is required.

### Expression of Chromophore-less phyB Restores phyC Levels as well as phyC Function in *phyB* Mutants

To address this question in detail, the PHYB derivative, PHYB(C364A) was used, where the chromophore attachment site, cysteine 364, was converted to alanine (Fig. 5A). The resulting molecule is expected to be inactive, as observed for PHYB(C357S) derivatives in *Arabidopsis* [12]. *PHYB(C364A)* or intact *PHYB* cDNA was introduced into *phyB* mutants, in which the *PHYA* allele is heterozygous to obtain *PHYB(C364A)/phyA phyB* (*PHYB(C364A)/aabb*) or *PHYB/phyA phyB* (*PHYB/aabb*) as segregated progenies. Immunoblot analyses showed that expression levels of PHYB(C364A) in the *PHYB(C364A)* lines and those of phyB in the *PHYB-*transgenic lines were comparable with those of WT in the etiolated seedlings (Fig. 5B). Notably, phyC levels were also increased in these transgenic lines corresponding to the increased levels of phyB, even if phyB is inactive. The resultant phyC levels were similar to those of WT (Fig. 5B). These results suggest that the expression of chromophore-less phyB restored phyC protein concentrations in the *phyB* mutant to levels comparable to WT.

To examine the biological activities of recovered phyC in the *PHYB* and *PHYB(C364A)* transgenic lines, coleoptile growth and *Lhcb* expression were analyzed in these transgenic lines. When grown in darkness, coleoptile lengths were not significantly different between the genotypes tested (Fig. 5C; Fig. S6A). Under FR, *phyA phyB* double mutants exhibited long coleoptiles similar to etiolated seedlings, but the expression of *PHYB* in the *phyA phyB* double mutants (*PHYB*/*aabb*) resulted in coleoptile lengths as short as those of *phyA* mutants (Fig. 5C; Fig. S6A). *Lhcb* genes (*Os03g0592500* and *Os09g0346500*) were also induced by FR in these *PHYB*/*aabb* transgenic seedlings (Fig. S6B). It is known that FR-mediated responses are attributed to only phyA or phyC in rice [20]. Therefore, recovered phyC, not introduced phyB, was responsible for de-etiolation of seedlings under FR in the *PHYB/aabb* lines, indicating that the phyC in *PHYB/aabb* transgenic lines is biologically active.

Before analyzing the biological activities of phyC in *PHYB(C364A*) transgenic lines, we first examined the activity of *PHYB(C364A)*. Takano et al. [20] reported that seedlings of *phyB* mutants exhibited a pale green phenotype under R. When seedlings of WT, *phyB*, *PHYB(C364A*)*/aabb*, and *PHYB/aabb* lines were grown under R for 8 days, WT and *PHYB/aabb* seedlings exhibited a dark green phenotype, while the *PHYB(C364A*) seedlings (#27-6 and #71-1) exhibited a pale green phenotype similar to that of *phyB* mutants (Fig. S6C). These observations indicate that *PHYB(C364A*) is insensitive to R in the transgenic lines. Thus, the *PHYB(C364A)/aabb* lines made it possible to analyze the real function of phyC in rice photomorphogenesis without the functional interference of phyA or phyB.

To examine the phyC-mediated responses to FR, seedlings from *PHYB(C364A*)*/Aabb* lines (#27-6) were grown under FR for 8 days (Fig. S6D). The coleoptile growth of *PHYB(C364A*)*/aabb* seedlings was significantly inhibited by FR, exhibiting dramatically shorter coleoptile lengths than those of *phyA phyB* double mutants (Fig. S6D, *PHYB(C364A*)*/aabb*). As shown in Figure 5D, the coleoptile growth was obviously inhibited by FR in all lines examined excluding the *phyA phyB* double mutant seedlings. Interestingly, the inhibition of coleoptile growth was more severe in *PHYB(C364A)/aabb* mutants than in *phyA* mutants, in both of which phyC is the only functional photoreceptor for FR (Fig. 5D). These results indicate that phyC is less sensitive to FR in the presence of functional phyB than in the presence of nonfunctional phyB. In addition, *PHYB(C364A)/Aabb* mutants had shorter coleoptiles compared with those of *phyB* mutants, which could be interpreted as the additive response of phyA and phyC to FR (Fig. 5D). Furthermore, expression of *Lhcb* genes (*Os03g0592500* and *Os09g0346500*) was also induced by FR in *PHYB(C364A)/aabb* seedlings (Fig. S6B, #27-6- and #71-1-). Thus, phyC can initiate downstream signaling only in the presence of phyB, even if phyB is spectrophotometrically inactive.

phyC-mediated responses to R in the inhibition of coleoptile growth and induction of light-regulated genes were also examined using *PHYB(C364A*) seedlings. When WT, *phyB*, *phyA phyB*, *PHYB(C364A)/aabb*, and *PHYB(C364A)/Aabb* seedlings were grown under R for 8 days, all seedlings, excluding those of *phyA phyB* double mutants, exhibited short coleoptiles (Fig. S6E). Moreover, the coleoptile lengths of *PHYB(C364A)/aabb* seedlings were as short as those of *PHYB(C364A*)*/Aabb* under R, and the coleoptile lengths of *PHYB(C364A)/aabb* seedlings were shorter than those of *phyB*. The differences were small but statistically significant (Fig. 5E). These results indicate that phyA and phyC respond to R in a highly redundant manner and that the phyC-mediated response to R is larger in magnitude than that of phyA, at least for the inhibition of coleoptile growth. In addition, *PHYB(C364A)/aabb* seedlings exhibited a pale green phenotype (Fig. S6E), indicating that phyC has a similar role to phyA in chlorophyll accumulation. Moreover, *Lhcb* genes were induced in *PHYB(C364A)/aabb* transgenic lines by R, and the expression levels of *Lhcb* genes were higher in *PHYB*(*C364A*)/*aabb* mutants than in *phyB* mutants (Fig. S6F, #27-6-, #69-2-, and #71-1-), which is consistent with the stronger inhibition of coleoptile growth in *PHYB*(*C364A*)/*aabb* mutants than in *phyB* mutants. These results indicate that phyC can sense R to control the de-etiolation of rice seedlings in the presence of phyB.

Collectively, phyC is biologically active in the presence of both functional and nonfunctional phyB protein, suggesting that the exertion of phyC function is not related to either the photo-sensing of phyB or the phyB-mediated responses but depends on complex formation with phyB. The co-IP assay revealed that phyC had a direct physical interaction with phyB in both *PHYB* and *PHYB*(*C364A*) transgenic seedlings (Fig. S5B).

These findings are different from the results obtained in *Arabidopsis*, in which expression of chromophore-less phyB restored phyC levels, but did not restore phyC activity [14]. It was concluded that phyC requires dimerization with chromophore-bearing phyB to have activity in *Arabidopsis*.

### Flowering Times of the Phytochrome Mutants

Under LD conditions (14L/10D), *phyB* and *phyC* mutants showed similar early flowering phenotypes, and *phyB phyC* double mutants flowered as early as *phyB* or *phyC* single mutants [20]. These observations suggest that phyB and phyC are probably equally involved in the control of flowering time for delaying response to LD conditions. However, we have not been able to exclude the possibility that phyB is not involved in the control of flowering times under the LD conditions because phyC is also dysfunctional in the *phyB* mutants. However, we can now overcome this limitation via *phyB*(*C364A*) mutants, which have functional phyA and phyC but dysfunctional phyB.

**Figure 6:**
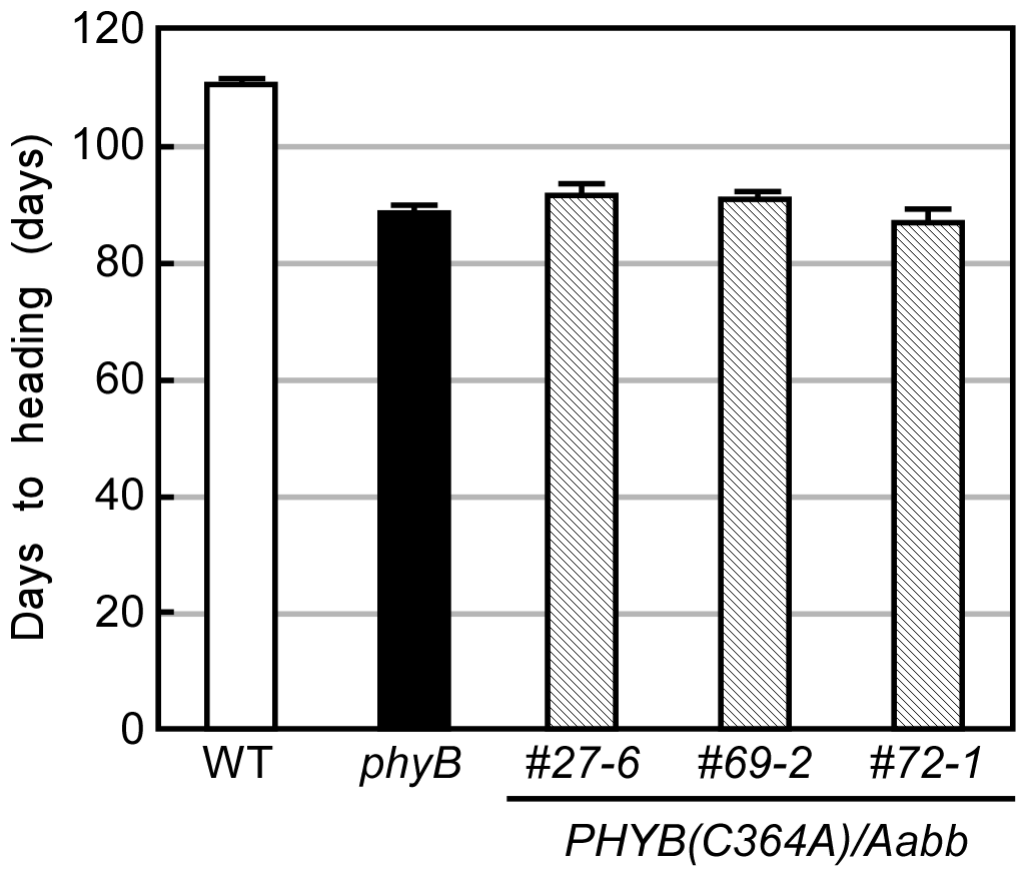
*PHYB(C364A)/Aabb* transgenic lines flower earlier than WT under LD conditions. Nipponbare (WT), *phyB-1* mutant (*phyB*), and *PHYB(C364A)*/*Aabb* transgenic lines were grown in a growth chamber set with LD (14.5L/9.5D) condition. The mean ± SE obtained from 20 plants is shown.

Three independent *phyB(C364A)* lines were grown under LD (14.5L/9.5D) conditions, and their flowering times were compared with those of WT and *phyB* mutants (Fig. 6). All *PHYB(C364A)/Aabb* lines flowered earlier than the WT by the same extent as the *phyB* mutant. These observations clearly confirmed that phyB and phyC are redundantly involved in the control of flowering time in response to LD conditions.

### phyB Is Involved in the Light-Induced Degradation of phyC in Rice

**Figure 7:**
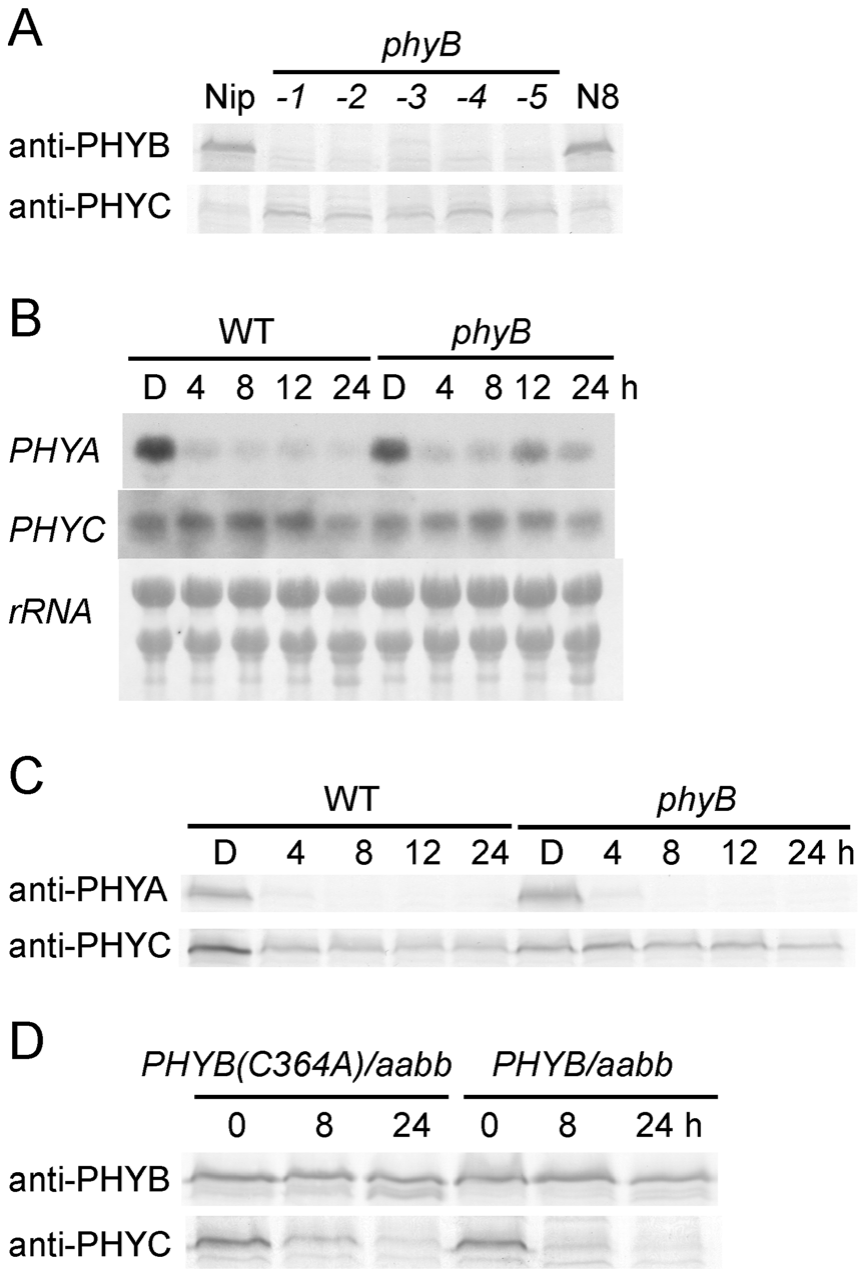
phyB is involved in light-induced degradation of phyC in rice. **A.** Levels of phyC are not reduced in *phyB* mutant seedlings grown under W. Protein extracts were prepared from WT and *phyB* seedlings grown under W for 7 days. Each lane was loaded with 50 µg of protein extracts for the detection of phyB and phyC using anti-PHYB and anti-PHYC antibodies, respectively. Nipponbare (Nip) and Norin8 (N8) were used as controls. *phyB-1*, *-2*, *-3*, *-4*, and *-5* are five different mutant alleles of *PHYB*. **B.** Effect of W on the transcript levels of *PHYA* and *PHYC* genes in WT and the *phyB* mutant. The seedlings of WT and *phyB-1* mutant (*phyB*) were grown for 5 days in the dark (D) and then exposed to W for 4, 8, 12, or 24 h before harvesting. For detecting the transcripts of *PHYA* and *PHYC*, each lane was loaded with 10 µg of total RNA. As a quantity control, rRNA was stained with methylene blue. **C.** Effect of W on phyA and phyC protein concentrations in WT and the *phyB* mutant. Growth conditions of the seedlings were the same as those in (**B**). Fifty micrograms of protein extract were loaded in each lane. **D.** PHYB(C364A) protein is necessary for the R-induced degradation of phyC. *PHYB(C364A)/aabb* (#27) and *PHYB*/*aabb* (#11) seedlings were grown in the dark (D) or in the D and then exposed to R for 8 or 24 h before harvesting. phyB and phyC proteins in 50 µg of protein extracts were detected with anti-PHYB and anti-PHYC antibodies, respectively.

In *Arabidopsis*, phyC concentrations have been shown to be lower in *phyB* mutants than in WT seedlings grown under dark as well as R conditions [23]–[25]. A similar phenomenon was observed in rice etiolated seedlings [20]. In the seedlings grown under W, however, phyC levels were similar in WT and *phyB* mutants (Fig. 7A). To address this issue more comprehensively, we examined the effect of W on both transcript and protein abundance of phyC at different time points after W irradiation in WT and *phyB* mutants. As shown in Figure 7B, *PHYA* mRNA levels were significantly down-regulated under these conditions, whereas transcript levels of *PHYC* remained relatively constant in WT seedlings as well as in *phyB* mutants, in accordance with the observation of Basu et al. [18]. These results suggest that the effect of W on the transcript level of *PHYC* is indistinguishable between WT and *phyB* mutants. Next, the light-stabilities of phyA and phyC proteins were examined in these seedlings. As shown in Figure 7C, protein abundance of phyA was rapidly reduced by W exposure in both WT and *phyB* mutants. On the other hand, phyC levels were reduced in WT but not in *phyB* mutants. As a result, the residual phyC levels in the *phyB* mutant were slightly higher than those in WT after being exposed to W for 24 h. These results imply that phyB protein is somehow involved in the light-induced degradation of phyC, but not phyA, in rice.

Next, we investigated the light-stability of restored phyC in *PHYB/aabb* and *PHYB(C364A)/aabb* transgenic lines. As shown in Figure 7D, the levels of phyC were obviously reduced by R irradiation in the seedlings of both *PHYB* and *PHYB(C364A)* mutants, while PHYB protein concentrations were nearly constant under these conditions in both seedlings. These results indicate that the restored phyC undergoes light-induced degradation. The formation of heterodimers between phyB and phyC is considered necessary for the light-induced selective degradation of phyC in rice.

It has been well established that the ubiquitin/26S proteasome pathway is involved in the Pfr-specific degradation of phyA in *Arabidopsis* [36]–[38] because a proteasome inhibitor, MG132, repressed the R-induced degradation of phyA in *Arabidopsis* [38]. Therefore, we tested the effect of MG132 on the R-induced degradation of phyC. Treatment with 50 µM MG132 significantly repressed the reduction of phyC and phyA in rice seedlings grown under R for 6 h (Fig. S7). These results suggest that the ubiqutin/26S proteasome pathway is involved in the light-induced degradation of phyC in rice seedlings, as is the case for *Arabidopsis* phyA.

## Discussion

### phyC Forms Heterodimers with phyB in Rice

In this study, it was revealed that all three native phytochrome proteins predominantly migrated at masses in the range of 316 to 404 kDa in rice etiolated seedlings (Fig. 2). These apparent masses are larger than the calculated 250 kDa for the dimeric forms of the phytochromes. However, similar observations were reported in *Arabidopsis* and oat. All five native *Arabidopsis* phytochrome proteins migrated at masses in the range of 300 to 380 kDa on SEC, and purified oat phyA had an apparent mass of 350 to 360 kDa on SEC, all of which are dimers [11], [13], [39]. Therefore, we conclude that native rice phytochromes also predominantly exist as dimers.

PhyB and phyC form heterodimeric complexes in *Arabidopsis* [13]. Native rice phyC was detected in dimer fractions on SEC using protein extracts from WT and *phyA* mutants and in monomer fractions in the absence of phyB both *in vivo* and *in vitro* (Fig. 2; Fig. S3D). Given the direct interaction between phyC and phyB (Figs. 1 and 2D), we deduced that phyC forms heterodimers with phyB in rice, similar to *Arabidopsis*. However, if phyB is absent, then phyC exists as a monomer. Even in its monomeric form, phyC is spectrophotometrically active (Fig. S3C), but biologically inactive.

The phyB to phyC ratio was estimated to be 1.3∶1 in etiolated seedlings (Fig. S1 and Supporting Information S1). Therefore, phyB exists as both phyB/phyC heterodimers and phyB/phyB homodimers in WT seedlings, and phyB homodimers and phyB/phyC heterodimers might have different roles in response to light. Microarray experiments using rice etiolated seedlings revealed that there is a cluster of genes that shows early and transient expression after an R-pulse in WT and *phyA* mutants, but not in *phyA phyC* mutants [40]. Because only phyB exists in the *phyA phyC* mutants, these results suggest that the early and transient gene expression induced by R is mediated by phyB/phyC heterodimers, but not by phyB homodimers.

### PHYB Protein is Essential for phyC-Mediated Responses in Rice

In this study, *PCG/aabb* seedlings exhibited the same phenotypes as *phyA phyB* seedlings, which do not respond to R and FR. In contrast, *PCG/aacc* seedlings responded to FR and showed de-etiolated phenotypes (Figs. 3C and 3D; Figs. S4A and S4B). These results indicate that phyC-GFP fusion proteins are biologically inactive in the *phyA phyB* background, but biologically active in the *phyA phyC* background, although in both cases higher phyC-GFP fusion protein concentrations were accumulated in the mutants compared to WT (Fig. 3B; Fig. S4C). Therefore, different phenotypes were observed depending on the absence or presence of phyB.

The molecular mechanism of the functional dependency of phyC on phyB was revealed by the experiments using *PHYB*(*C364A*)/*aabb* transgenic lines, in which chromophore-less PHYB was expressed, and phyC levels were recovered to those observed in the WT. These seedlings responded to both R and FR, which indicates that the functional rescue of phyC in *PHYB*(*C364A*)/*aabb* is attributed to the presence of phyB, even when it is non-functional, but not to the increased levels of phyC because phyC levels are significantly lower in *PHYB*(*C364A*)/*Aabb* transgenic lines than phyC-GFP levels in *PCG/aabb* mutants (Figs. 3B and 5B). Furthermore, it became clear for the first time that phyC is involved in the responses to both FR and R for the inhibition of coleoptile growth as well as the induction of *Lhcb* genes in rice (Fig. 5; Fig. S6).

It is worth noting that *PCG/aacc* mutants exhibited the same phenotypes as *phyA* mutants under FR despite the significantly higher abundance of the phyC-GFP fusion protein in these transgenic seedlings compared to those in *phyA* seedlings (Fig. 3). This suggests that the limiting factor is the level of phyB, which contributes to the formation of phyB/phyC heterodimers in *PCG/aacc* seedlings. In turn, this observation provides indirect evidence that the heterodimeric complex of phyB/phyC is the only functional form for the phyC-mediated responses to light.

### Photosensory Specificities of phyC in Rice

As Clack et al. [14] mentioned, heterodimerization of phyB and phyC is likely fundamental throughout plants. However, there is a difference between *Arabidopsis* and rice. *Arabidopsis* phyC requires dimerization with functional phyB to have activity [14], while rice phyC is active even while forming heterodimers with chromophore-less PHYB. This difference seems to be correlated with the functional differentiation of phyC during evolution. Rice phyC is involved in the responses to both R and FR in the presence of phyB (Fig. 5; Fig. S6). Thus, the photosensory specificity of phyC is similar to that of phyA and different from that of phyB in rice. In contrast, *Arabidopsis* phyC does not participate in the control of seedling de-etiolation under FR, but does participate in controlling responses to R [23], [41].

Regarding the inhibition of coleoptile growth under R, the inhibitory effect mediated by phyC was similar to that mediated by phyA, as demonstrated by the fact that *PHYB*(*C364A*)/*aabb* seedlings exhibited the same phenotype as *PHYB*(*C364A*)/*Aabb* seedlings (Fig. 5E). In addition, the seedlings of *phyA* and *phyC* mutants did not show any different phenotypes from those of WT under R [20]. These observations indicate that phyA, phyB, and phyC act in a highly redundant manner to control coleoptile growth under R.

phyA is the principal photoreceptor for FR, while phyC plays a minor role, because *phyA* mutants show more pronounced phenotypes than *phyC* mutants under FR [20]. Unexpectedly, however, *PHYB*(*C364A*)/*aabb* seedlings exhibited significantly shorter coleoptiles than *phyA* seedlings under FR, although phyC is the only photoreceptor for FR in these two genotypes (Fig. 5D). These findings may indicate that phyB has an antagonistic function against the phyC-mediated responses to FR. When phytochrome molecules are activated to their Pfr forms, a “Z” to “E” isomerization occurs in the C-15 double bond between the C and D rings of the linear tetrapyrrole [42]. The Pfr chromophore is in a distorted, high-energy C15-E anticonfiguration. Chromophore-apoprotein interactions (including the structural relaxation at the chromophore-binding site) and subsequent conformational changes in the protein backbone are required to maintain the high-energy state [42]–[45]. In this context, when phyB/phyC heterodimers are exposed to FR, phyC subunits, similar to *Arabidopsis* phyA, absorb FR and adopt the phyB Pr/phyC Pfr configuration because phyB Pr cannot sense FR [20]. Pr-conformation of phyB subunits may prevent phyC subunits from maintaining the high-energy Pfr form, whereas *phyB*(*C364A*) subunits lacking the chromophore may not affect the Pfr conformation of phyC subunits.

Hening and Schäfer [46] prepared *Arabidopsis* phyA dimers that incorporate the essential chromophore only in one subunit by using a coexpression system in yeast and demonstrated that such mixed dimers showed unaltered difference spectra. Their results, together with our findings, indicate that the absence of a chromophore from one subunit of a phytochrome dimer does not affect the photoreversible change of the functional phytochrome.

### phyB Is Involved in the Flowering Time Determination under LD

For the involvement of phytochromes in the regulation of flowering time, phyB has been considered responsible for delaying flowering in response to LD conditions because *phyB* mutants flower earlier than WT under LD conditions [20]. However, because *phyB*, *phyC*, and *phyB phyC* mutants all flowered earlier than WT in LD [20], and because phyC is also dysfunctional in the *phyB* mutants, the results for the *phyB* mutants must be carefully reconsidered. In this report, we created *PHYB(C364A)*/*Aabb* transgenic rice, in which only phyB is dysfunctional, and demonstrated that phyB is surely involved in the control of flowering time by suppressing the floral initiation under LD conditions in rice (Fig. 6). Moreover, the observations that *phyB*, *phyC*, and *phyB phyC* mutants similarly flowered earlier than WT in LD can be easily explained if phyB/phyC heterodimers are responsible for the suppression of flowering under LD.

### phyB Affects the Stability of phyC in Both Etiolated and Light-grown Rice Seedlings

PhyC levels are decreased in *phyB* mutants in rice, which is attributed to a posttranslational event because RNA blot analyses of *PHYC* did not show any differences between *phyB* mutants and WT [20]. In this study, chromophore-less phyB recovered phyC to WT levels (Fig. 5B), suggesting that phyB is indispensable for stabilizing phyC by forming phyB/phyC heterodimers in etiolated seedlings.

RNA blot and immunoblot analyses suggest that phyB is necessary for light-induced degradation of phyC by formation of phyB/phyC heterodimers (Fig. 7). Recently, several studies about the degradation of *Arabidopsis* phyA have revealed that light-induced degradation of phyA is in part controlled by light-regulated import into the nucleus where the turnover rate is significantly faster than in the cytoplasm [47], [48]. Based on these observations, we speculate that the rice phyC monomer is probably unable to undergo nuclear translocation, which imparts stability.

**Figure 8:**
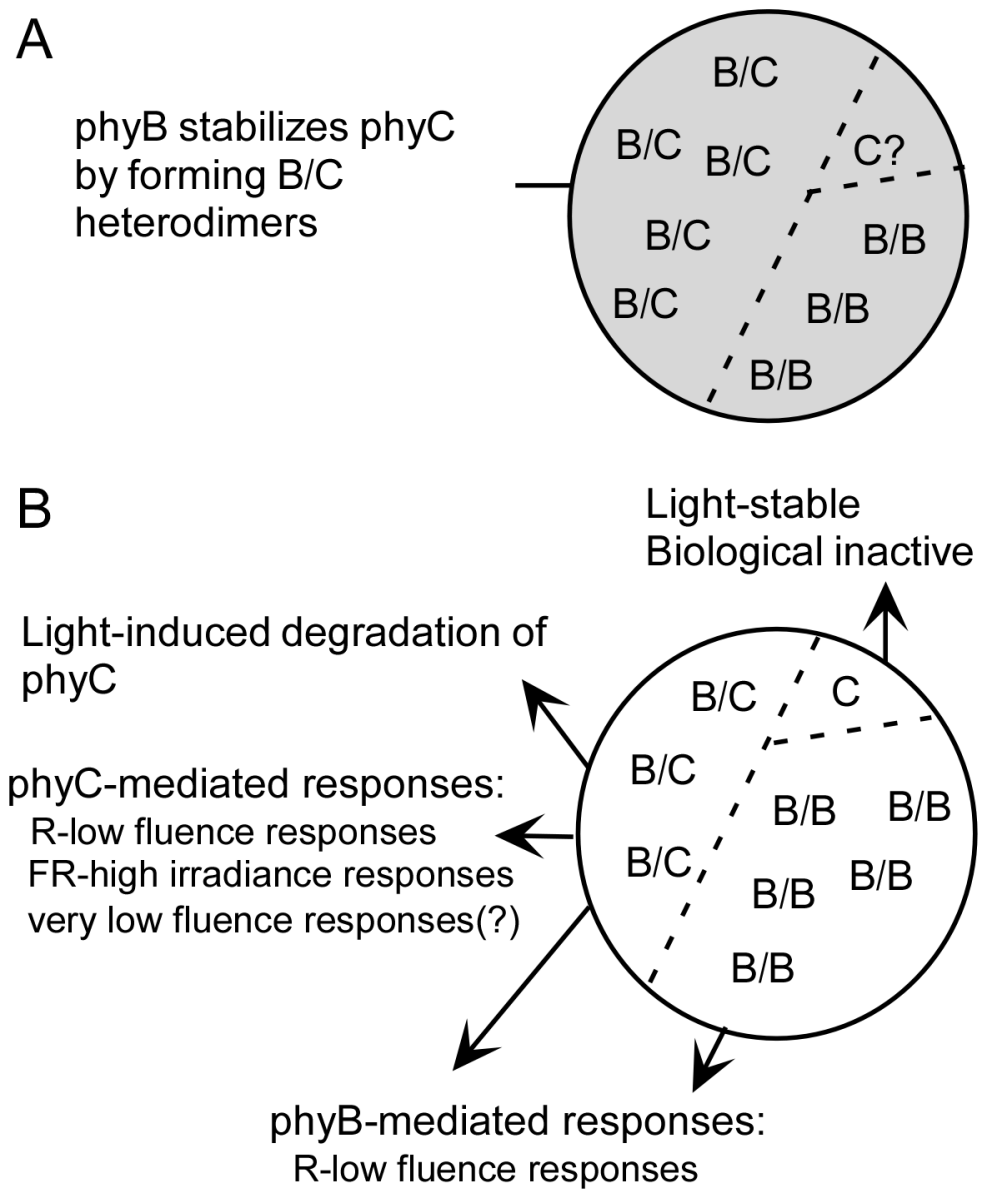
State models of phyC and phyB in rice seedlings. **A.** In the cells of WT seedlings grown in the dark, most of the phyC exists as phyB/phyC heterodimers (B/C) and, probably, to a smaller degree as phyC monomers (C?), while phyB exist as phyB/phyC heterodimers (B/C) and phyB/phyB homodimers (B/B). In etiolated seedlings, phyB stabilizes phyC in the B/C conformation. Consequently, phyC levels are quite low in the *phyB*-deficient mutants. **B.** When seedlings are exposed to light, including W, R, and FR, phyC subunits in phyB/phyC heterodimers (B/C) are light-labile and biologically active to participate in the multiple processes of rice development (inhibition of coleoptile growth, induction of light-regulated genes, and chlorophyll accumulation). By contrast, phyC monomers (C) are light-stable but do not participate in the de-etiolation of rice seedlings. phyB in its heterodimeric (B/C) and homodimeric (B/B) forms probably play different roles in the responses to light.

Treatment of etiolated seedlings with MG132 delayed light-induced degradation of phyC (Fig. S7), suggesting that the ubiquitin/26S proteasome pathway is involved in phyC degradation steps as observed for *Arabidopsis* phyA. For light-induced degradation of *Arabidopsis* phyA, multiple ubiquitins are covalently attached to the phyA Pfr; these ubiquitin-protein conjugates then serve as substrates for the 26S proteasome complex [36]–[38], [49]. Regarding rice phyC, we hypothesize that formation of the phyB/phyC heterodimer is necessary for nuclear translocation to transduce the signal and allow phyC to undergo ubiquitination (Fig. 8). It should also be noted that light irradiation in etiolated seedlings caused concentrations of phyC, but not phyB, to decrease. We speculate that the phyC subunit is a selective target of ubiquitination in the phyB/phyC heterodimer and that after degradation of phyC, the remaining phyB subunits likely form homodimers.

In summary, our data indicate that phyC predominantly exists as phyB/phyC heterodimers in etiolated seedlings (Fig. 8A). As such, the amount of phyC in *phyA phyB* mutants is greatly reduced, and the remaining phyC exists in monomeric form and has no function (Fig. 8A). Homodimer formation of phyC has not been observed in *Arabidopsis* [13], [14]. Therefore, formation of the phyB/phyC heterodimer stabilizes phyC and is indispensable for the light-induced function of phyC in the control of the de-etiolation of rice seedlings and for the light-induced degradation of phyC (Fig. 8B). Physical interaction between phyB and phyC has been conserved in both *Arabidopsis* and rice, but the function of phyC has been differentiated during evolution. Recent progress in the structure–function relationship of phytochromes, which extensively revealed the function of the various domains of phytochrome molecules in light-dependent signaling [50]–[52], will provide clues for explaining the functional differentiations of phyC in future.

## Supporting Information

Figure S1 **Quantification of relative phyB and phyC concentrations in the protein extracts of rice seedlings.** **A.** CBB staining of PHYB-His and PHYC-His proteins. The purified PHYB-His and PHYC-His proteins were separated by 12% SDS-PAGE and stained by CBB R-250. The signal intensities were analyzed using NIH image 1.62. The loaded amounts of proteins were 15, 10, 7.5, 5.0, and 2.5 µl for PHYB-His and PHYC-His. **B.** Immunoblots of phyB and PHYB-His proteins. Protein extracts from 5-day-old etiolated WT seedlings and dilution series of PHYB-His standard proteins were separated by SDS-PAGE in the same gel. PhyB was detected using anti-PHYB antibody. The loaded amounts of proteins were 50, 25, 12.5, and 6.3 µg for detecting phyB proteins in the protein extracts. The loaded amounts of proteins were 13, 6.5, and 3.2 µl of 1000× diluted purified PHYB-His protein for quantifying standard PHYB-His protein. The signal intensities were analyzed using NIH image 1.62. **C.** Immunoblots of phyC and PHYC-His proteins. Protein extracts from 5-day-old etiolated WT seedlings and dilution series of PHYB-His standard proteins were separated by SDS-PAGE in the same gel. PhyC was detected using anti-PHYC antibody. The loaded amounts of proteins were 50, 25, 12.5, and 6.3 µg for detecting phyC protein in the protein extracts. The loaded amounts of proteins were 13, 6.5, and 3.2 µl of 1000× diluted purified PHYC-His protein for quantifying standard PHYC-His proteins. The signal intensities were analyzed using NIH image 1.62.
(TIF)

Figure S2 **Immunoblot analyses of phyB and phyC light-stabilities in rice seedlings. A.** Effect of W on phyB and phyC levels in the WT seedlings. The WT seedlings were grown in the dark (D) for 6 days or in the dark for 6 days and then exposed to W for 0.5, 1, 2, 4, 8, 12, or 24 h before harvesting. Protein extracts were prepared from these seedlings. Fifty micrograms of protein extract were loaded for detecting phyB and phyC with anti-PHYB and anti-PHYC antibodies, respectively. Relative signal intensities of protein bands were analyzed using Gel-Pro Analyzer 4.0 software (Media Cybernetics, USA). **B.** Dilution series of protein extracts from the seedlings grown in the dark (D) were compared with the protein extracts from the seedlings exposed to W for 24 h.
(TIF)

Figure S3 **Difference spectra and SEC profiles of recombinant phyA and phyC proteins expressed in** ***E. coli*** **. A.** Schematic drawing of a phytochrome molecule and a construction for expression of full length phytochromes (for both phyA and phyC). For the purification, CBP (calmodulin-binding peptide) is attached at N-terminal and 6× His at C-terminal. Native chromophore, phytochromobilin is used. **B** and **C.** Absorbance spectra (left) and R/FR difference spectrum (right) of recombinant rice phyA (**B**) and phyC (**C**). **D.** Recombinant proteins expressed in *E. coli* were fractionated by SEC and phyA and phyC proteins were immunochemically detected with anti-His tag (upper) or anti-phytochrome antibodies (lower) in the individual fractions (#17–#24). Small numbers above the fraction numbers are the molecular sizes which were calculated based on the calibration line of standard proteins.
(TIF)

Figure S4 **phyC-GFP is biologically active in** ***phyA phyC*** **backgrounds and inactive in** ***phyB-*** **deficient backgrounds.** **A.** Visual phenotypes of WT, *phyA*, *phyA phyC*, and *PCG/aacc* seedlings (#19-1) grown under D or FR for 8 days. White arrow heads indicate apices of coleoptiles in the seedlings grown under FR. Bar  = 10 mm. **B.** FR-induced expression of *Lhcb* genes in *PCG/aacc* transgenic seedlings. WT, *phyA*, *phyA phyC*, and *PCG/aacc* seedlings (#19-1 and #20-1) grown under D or FR for 7 days. Transcript levels of two *Lhcb* genes (*Os03g0592500* and *Os09g034650*) were analyzed by RT-PCR. Ubiquitin (*UBQ*) was used as an internal control. **C.** Visual phenotypes of WT, *phyB*, *phyA phyB*, and *PCG/Aabb* (#7-4-) seedlings grown under D or FR for 8 days. Segregated *PCG/aabb* genotypes were identified by genotyping PCR. The abundance of phyC-GFP fusion proteins was compared between *PCG/Aabb* and *PCG/aabb* mutants by immunoblot analysis. White arrow heads indicate apices of coleoptiles in the *PCG* transgenic seedlings grown under FR. Bar  = 10 mm. **D.** FR could not induce the expression of *Lhcb* genes in *PCG/aabb* transgenic seedlings. WT, *phyB*, *phyA phyB* and *PCG/aabb* seedlings (#23 and #32) grown under D or FR for 7 days. Transcript levels of two *Lhcb* genes (*Os03g0592500* and *Os09g034650*) were analyzed by RT-PCR. Ubiquitin (*UBQ*) was used as an internal control. **E.** Visual phenotypes of WT, *phyB*, *phyA phyB*, and *PCG/Aabb* (#12-5-) and segregated *PCG/aabb* seedlings grown under D or R for 8 days. White arrow heads indicate apices of coleoptiles in the *PCG* transgenic seedlings grown under R. Bar  = 10 mm. **F.** R could not induce the expression of *Lhcb* genes in *PCG/aabb* transgenic seedlings (#7-4 and #12-5).
(TIF)

Figure S5 **A physical interaction exists between phyB and phyC in overexpresser lines of** ***PHYC-GFP*** **,** ***PHYB*** **, and** ***PHYB*** **(** ***C364A*** **).** **A.** Co-IP assay of phyC-GFP and phyB in *PCG/aacc* seedlings. The protein extracts from 7-day-old etiolated seedlings of *PCG*/*aacc* (#5, #15, #19, #7, and #8) were immunoprecipitated with anti-PHYC antibody. PhyB and phyC-GFP were detected by immunoblot analyses. Thirty micrograms of protein extracts from PCG/aacc #7 were loaded as the positive control (Ext). **B.** Co-IP assay of phyC and phyB in *PHYB* and *PHYB(C364A)* transgenic seedlings. The protein extracts from 7-day-old etiolated seedlings of *PHYB/aabb* (#5 and #7) and *PHYB(C364A)*/*aabb* (#27 and #62) mutants were immunoprecipitated with anti-PHYC antibody. PhyB and phyC were detected by immunoblot analyses. Thirty micrograms of protein extracts from WT seedlings were loaded as the positive control (Ext).
(TIF)

Figure S6 **phyC is biologically active in** ***PHYB*** **and** ***PHYB(C364A)*** **transgenic lines.** **A.** Visual phenotypes of WT, *phyA*, *phyA phyB*, and *PHYB*/*aabb* (#7-2- and #11-3-) seedlings grown in the dark (D) or under FR (FR) for 8 days. White arrow heads indicate the apices of coleoptiles. Bar  = 10 mm. **B.** FR-induced expression of *Lhcb* genes in *PHYB*/*aabb* and *PHYB(C364A)*/*aabb* transgenic seedlings. WT, *phyB*, *phyA phyB*, *PHYB*/*aabb* (#7-2- and #11-3-), and *PHYB(C364A)*/*aabb* (#27-6- and #71-1-) seedlings grown for 7 days. Transcript levels of two *Lhcb* genes (*Os03g0592500* and *Os09g034650*) were analyzed by RT-PCR. Ubiquitin (*UBQ*) was used as an internal control. **C.** *PHYB(C364A)* transgenic lines and *phyB* mutants exhibited a pale green phenotype under R. Visual phenotypes of WT, *phyB*, *phyA phyB*, two lines of *PHYB(C364A)/aabb* (#27-6- and #71-2-), and two lines of *PHYB*/*aabb* (#7-1- and #11-3-) seedlings grown under R for 7 days. Bar  = 10 mm. **D** and **E.** Visual phenotypes of WT, *phyB*, *phyA phyB*, and *PHYB(C364A)*/*Aabb* (#27-6-) seedlings grown under FR (**D**) or R (**E**) for 8 days. Mutated and wild-type *PHYA* alleles are indicated by *a* and *A*, respectively. White arrow heads in (**D**) and (**E**) indicate the apices of coleoptiles of 8-day old seedlings. Bar  = 10 mm. **F.** R-induced expression of *Lhcb* genes in the *PHYB(C364A)*/*aabb* seedlings. Transcript levels of two *Lhcb* genes (*Os03g0592500* and *Os09g034650)* were analyzed by RT-PCR in the *PHYB(C364A)*/*aabb* seedlings (#27-6- #69-2-, and #71-1-). Ubiquitin (*UBQ*) was used as an internal control.
(TIF)

Figure S7 **Treatment with MG132 delays light-induced degradation of phyA and phyC in rice seedlings.** Four-day-old etiolated WT seedlings (D) were treated with 50 µM MG132 (+) or 0.5% DMSO (−) for 1.5 h and then exposed to R for 0 or 6 h before harvesting. Protein extracts (50 µg) from these seedlings were used to detect phyA and phyC with monoclonal anti-rye PHYA (mAR08) and anti-PHYC antibodies, respectively.
(TIF)

Supporting Information S1 **Quantification of relative phyB and phyC protein concentrations in rice seedlings.** To establish the calibration curves of purified proteins, the purified proteins were separated by SDS-PAGE and stained with CBB R-250. Signal intensities of CBB-stained purified protein bands were analyzed using NIH image 1.62 software for PHYB-His and PHYC-His (Fig. S1A). The graph (A) was produced using the loaded amounts of proteins as X-axes and the signal intensities of CBB-stained PHYB-His or PHYC-His standard proteins as Y-axes. The regression lines were fitted as Y = 61.9X for PHYB-His and Y = 78X for PHYC-His. Therefore, given the loaded amounts of the standard proteins, the corresponding intensities of CBB-stained PHYB-His or PHYC-His were calculated according to the regression lines. For quantifying the relative levels of phyB and phyC proteins, protein extracts from 5-day-old etiolated seedlings and dilution series of standard proteins (PHYB-His or PHYC-His, they were diluted by 1000-fold for immunodetection) were separated by SDS-PAGE in the same gel. phyB and phyC were immunochemically detected with anti-PHYB and anti-PHYC antibodies, respectively. Signals intensities of the representative immunoblot for phyB (or phyC) and PHYB-His (or PHYC-His) were scanned and analyzed using NIH image 1.62 software (Figs. S1B and S1C). Because the signal intensities of immunoblots with different antibodies cannot be compared directly. They were converted into the intensities of CBB staining using the data from standard proteins (PHYB-His or PHYC-His). The graph (B) was plotted with the signal intensities of CBB-stained PHYB-His or PHYC-His proteins as the X-axis and signal intensities of immunoblots as the Y-axis. The regression line was fitted. Thus, the corresponding CBB-stained intensity per immunoblot signal for phyB and phyC in the protein extracts were calculated. Then, the graph (C) was plotted with the loaded amount of protein extracts as the X-axis and the calculated CBB signals as the Y-axis. The linear regression lines of Y = 16.7X and Y = 13.2X were obtained for phyB and phyC proteins, respectively. Therefore, the ratio of phyB:phyC was 1.3∶1 in the dark-grown WT seedlings.
(PDF)
