# Supplementary figures and images for: The Phytochrome B/Phytochrome C Heterodimer Is Necessary for Phytochrome C-Mediated Responses in Rice Seedlings
Source: PLoS One. 2014 May 22;9(5):e97264. doi: 10.1371/journal.pone.0097264 (PMC4031084; doi:10.1371/journal.pone.0097264)

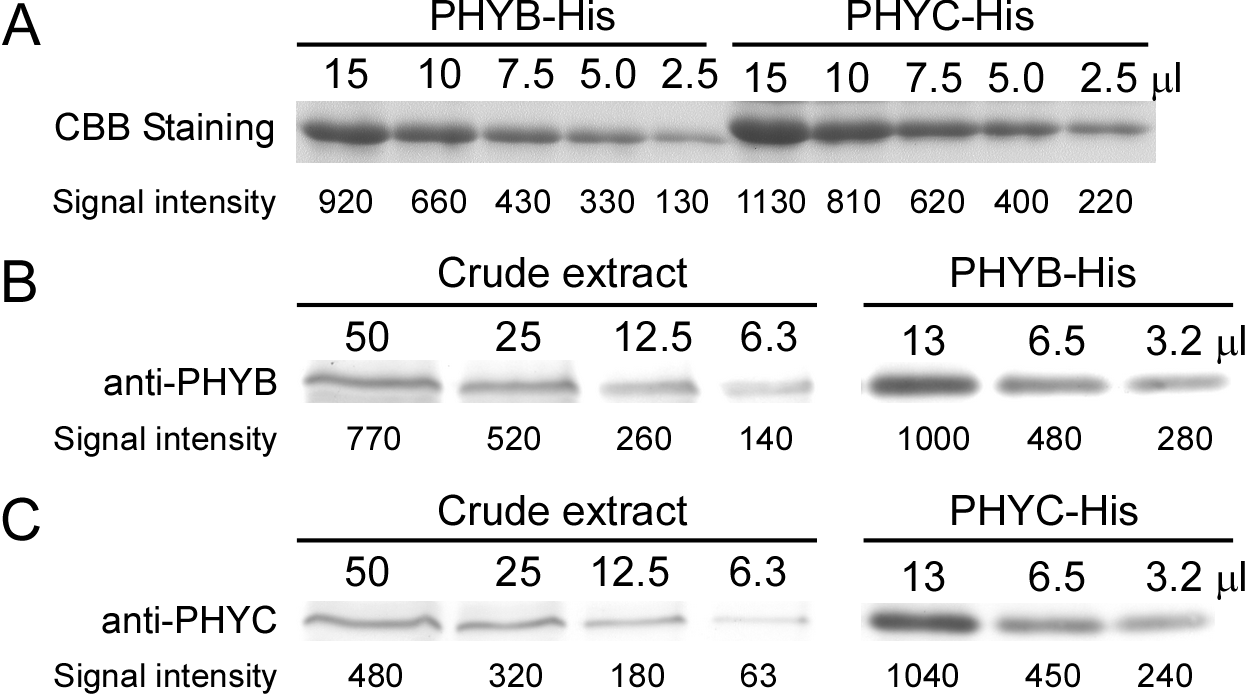

Supplement: Figure S1 — Quantification of relative phyB and phyC concentrations in the protein extracts of rice seedlings. A. CBB staining of PHYB-His and PHYC-His proteins. The purified PHYB-His and PHYC-His proteins were separated by 12% SDS-PAGE and stained by CBB R-250. The signal intensities were analyzed using NIH image 1.62. The loaded amounts of proteins were 15, 10, 7.5, 5.0, and 2.5 µl for PHYB-His and PHYC-His. B. Immunoblots of phyB and PHYB-His proteins. Protein extracts from 5-day-old etiolated WT seedlings and dilution series of PHYB-His standard proteins were separated by SDS-PAGE in the same gel. PhyB was detected using anti-PHYB antibody. The loaded amounts of proteins were 50, 25, 12.5, and 6.3 µg for detecting phyB proteins in the protein extracts. The loaded amounts of proteins were 13, 6.5, and 3.2 µl of 1000× diluted purified PHYB-His protein for quantifying standard PHYB-His protein. The signal intensities were analyzed using NIH image 1.62. C. Immunoblots of phyC and PHYC-His proteins. Protein extracts from 5-day-old etiolated WT seedlings and dilution series of PHYB-His standard proteins were separated by SDS-PAGE in the same gel. PhyC was detected using anti-PHYC antibody. The loaded amounts of proteins were 50, 25, 12.5, and 6.3 µg for detecting phyC protein in the protein extracts. The loaded amounts of proteins were 13, 6.5, and 3.2 µl of 1000× diluted purified PHYC-His protein for quantifying standard PHYC-His proteins. The signal intensities were analyzed using NIH image 1.62. (TIF) [file pone.0097264.s001.tif]

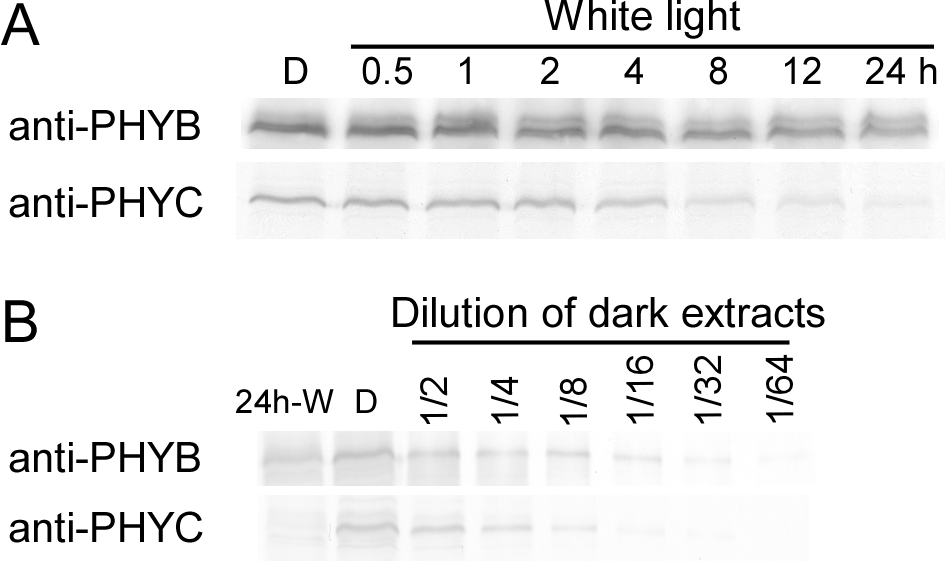

Supplement: Figure S2 — Immunoblot analyses of phyB and phyC light-stabilities in rice seedlings. A. Effect of W on phyB and phyC levels in the WT seedlings. The WT seedlings were grown in the dark (D) for 6 days or in the dark for 6 days and then exposed to W for 0.5, 1, 2, 4, 8, 12, or 24 h before harvesting. Protein extracts were prepared from these seedlings. Fifty micrograms of protein extract were loaded for detecting phyB and phyC with anti-PHYB and anti-PHYC antibodies, respectively. Relative signal intensities of protein bands were analyzed using Gel-Pro Analyzer 4.0 software (Media Cybernetics, USA). B. Dilution series of protein extracts from the seedlings grown in the dark (D) were compared with the protein extracts from the seedlings exposed to W for 24 h. (TIF) [file pone.0097264.s002.tif]

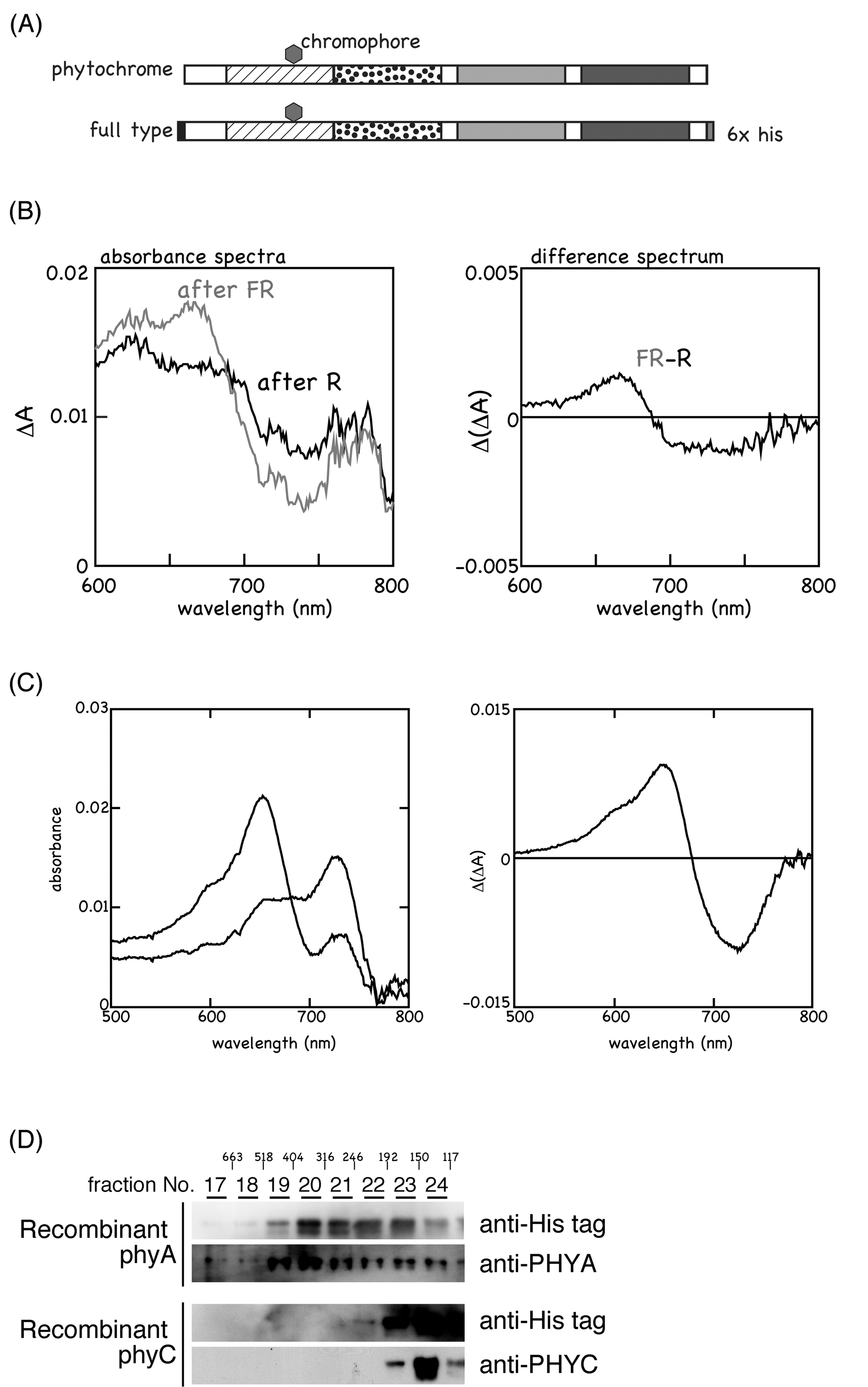

Supplement: Figure S3 — Difference spectra and SEC profiles of recombinant phyA and phyC proteins expressed in E. coli . A. Schematic drawing of a phytochrome molecule and a construction for expression of full length phytochromes (for both phyA and phyC). For the purification, CBP (calmodulin-binding peptide) is attached at N-terminal and 6× His at C-terminal. Native chromophore, phytochromobilin is used. B and C. Absorbance spectra (left) and R/FR difference spectrum (right) of recombinant rice phyA (B) and phyC (C). D. Recombinant proteins expressed in E. coli were fractionated by SEC and phyA and phyC proteins were immunochemically detected with anti-His tag (upper) or anti-phytochrome antibodies (lower) in the individual fractions (#17–#24). Small numbers above the fraction numbers are the molecular sizes which were calculated based on the calibration line of standard proteins. (TIF) [file pone.0097264.s003.tif]

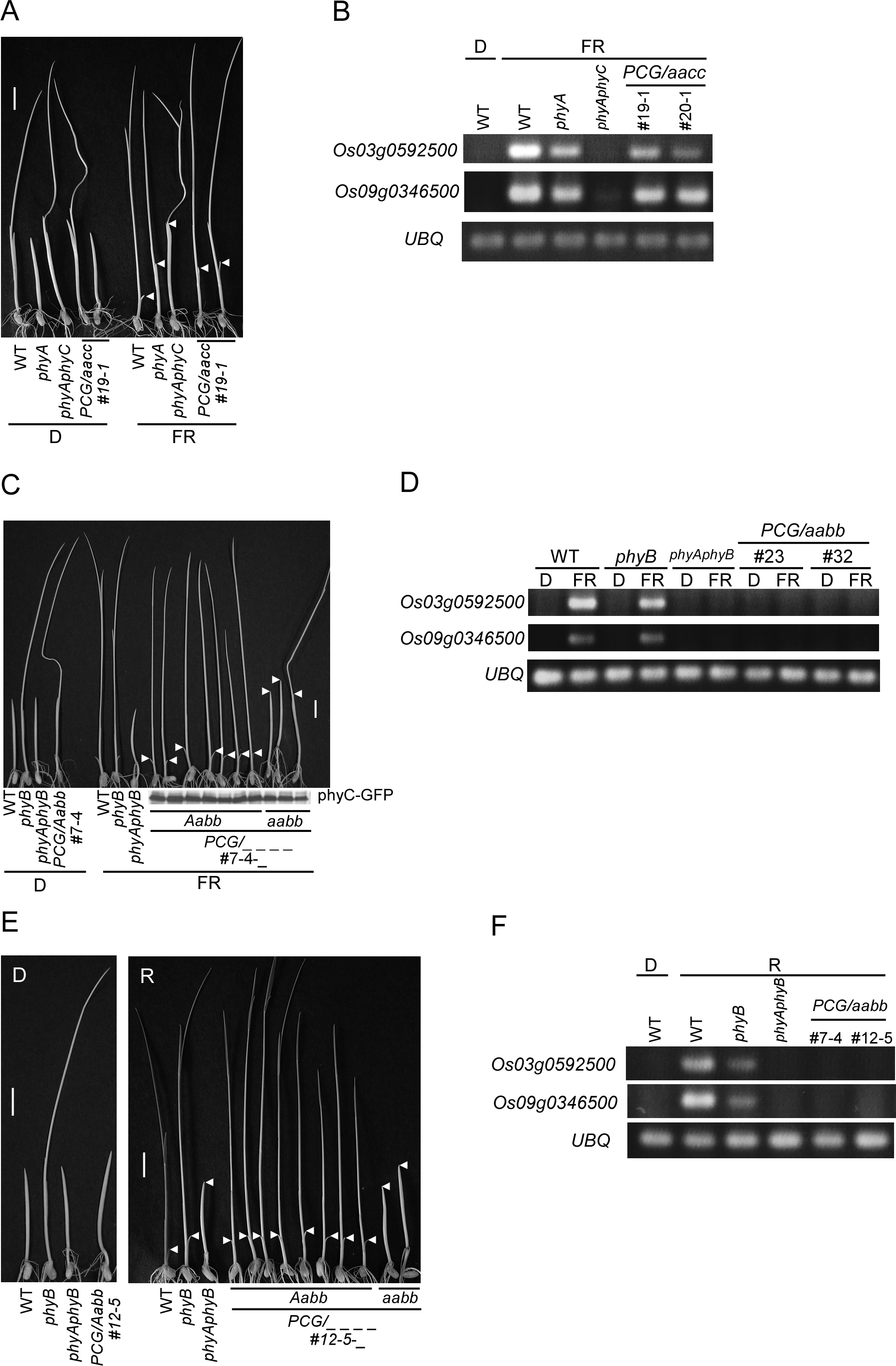

Supplement: Figure S4 — phyC-GFP is biologically active in phyA phyC backgrounds and inactive in phyB- deficient backgrounds. A. Visual phenotypes of WT, phyA, phyA phyC, and PCG/aacc seedlings (#19-1) grown under D or FR for 8 days. White arrow heads indicate apices of coleoptiles in the seedlings grown under FR. Bar = 10 mm. B. FR-induced expression of Lhcb genes in PCG/aacc transgenic seedlings. WT, phyA, phyA phyC, and PCG/aacc seedlings (#19-1 and #20-1) grown under D or FR for 7 days. Transcript levels of two Lhcb genes (Os03g0592500 and Os09g034650) were analyzed by RT-PCR. Ubiquitin (UBQ) was used as an internal control. C. Visual phenotypes of WT, phyB, phyA phyB, and PCG/Aabb (#7-4-) seedlings grown under D or FR for 8 days. Segregated PCG/aabb genotypes were identified by genotyping PCR. The abundance of phyC-GFP fusion proteins was compared between PCG/Aabb and PCG/aabb mutants by immunoblot analysis. White arrow heads indicate apices of coleoptiles in the PCG transgenic seedlings grown under FR. Bar = 10 mm. D. FR could not induce the expression of Lhcb genes in PCG/aabb transgenic seedlings. WT, phyB, phyA phyB and PCG/aabb seedlings (#23 and #32) grown under D or FR for 7 days. Transcript levels of two Lhcb genes (Os03g0592500 and Os09g034650) were analyzed by RT-PCR. Ubiquitin (UBQ) was used as an internal control. E. Visual phenotypes of WT, phyB, phyA phyB, and PCG/Aabb (#12-5-) and segregated PCG/aabb seedlings grown under D or R for 8 days. White arrow heads indicate apices of coleoptiles in the PCG transgenic seedlings grown under R. Bar = 10 mm. F. R could not induce the expression of Lhcb genes in PCG/aabb transgenic seedlings (#7-4 and #12-5). (TIF) [file pone.0097264.s004.tif]

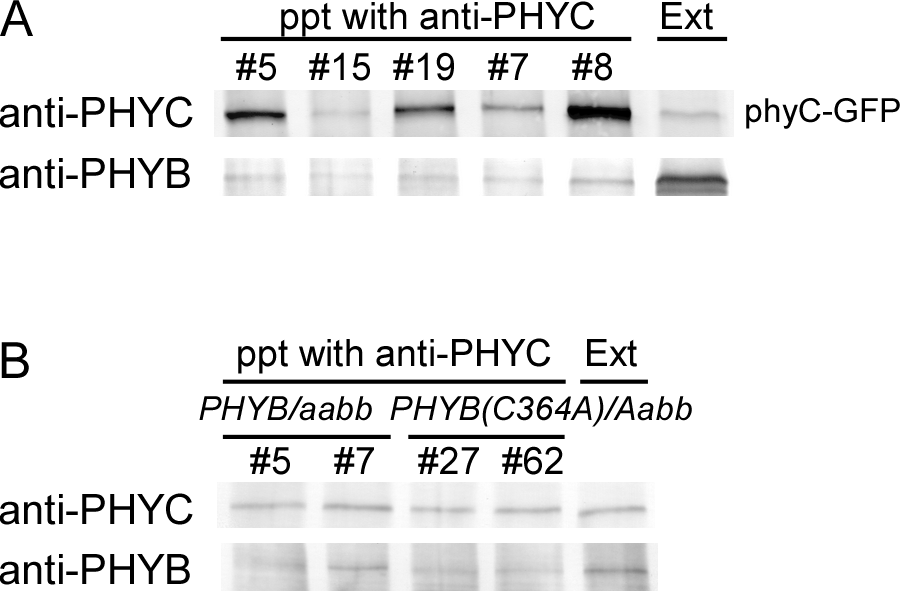

Supplement: Figure S5 — A physical interaction exists between phyB and phyC in overexpresser lines of PHYC-GFP , PHYB , and PHYB ( C364A ). A. Co-IP assay of phyC-GFP and phyB in PCG/aacc seedlings. The protein extracts from 7-day-old etiolated seedlings of PCG/aacc (#5, #15, #19, #7, and #8) were immunoprecipitated with anti-PHYC antibody. PhyB and phyC-GFP were detected by immunoblot analyses. Thirty micrograms of protein extracts from PCG/aacc #7 were loaded as the positive control (Ext). B. Co-IP assay of phyC and phyB in PHYB and PHYB(C364A) transgenic seedlings. The protein extracts from 7-day-old etiolated seedlings of PHYB/aabb (#5 and #7) and PHYB(C364A)/aabb (#27 and #62) mutants were immunoprecipitated with anti-PHYC antibody. PhyB and phyC were detected by immunoblot analyses. Thirty micrograms of protein extracts from WT seedlings were loaded as the positive control (Ext). (TIF) [file pone.0097264.s005.tif]

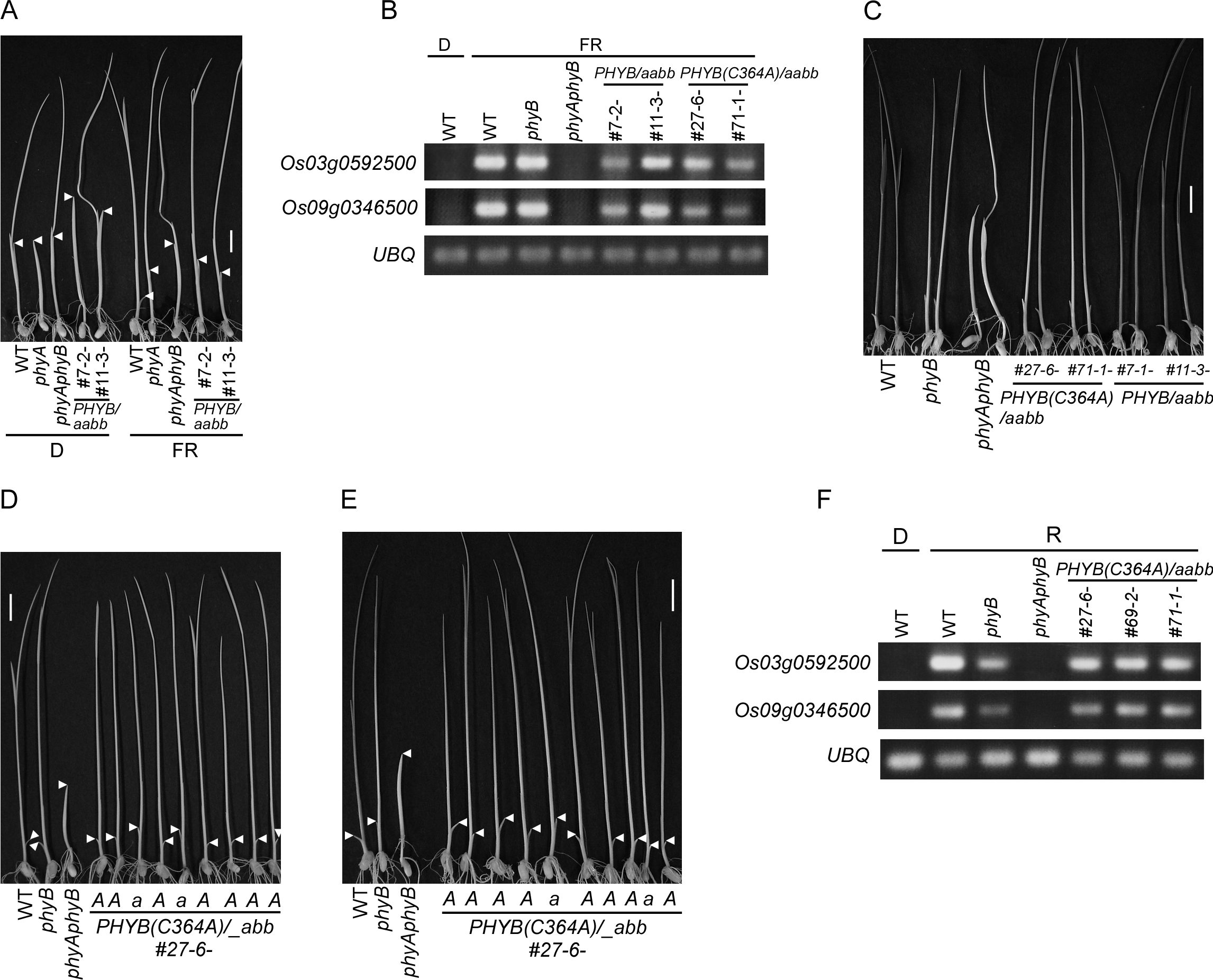

Supplement: Figure S6 — phyC is biologically active in PHYB and PHYB(C364A) transgenic lines. A. Visual phenotypes of WT, phyA, phyA phyB, and PHYB/aabb (#7-2- and #11-3-) seedlings grown in the dark (D) or under FR (FR) for 8 days. White arrow heads indicate the apices of coleoptiles. Bar = 10 mm. B. FR-induced expression of Lhcb genes in PHYB/aabb and PHYB(C364A)/aabb transgenic seedlings. WT, phyB, phyA phyB, PHYB/aabb (#7-2- and #11-3-), and PHYB(C364A)/aabb (#27-6- and #71-1-) seedlings grown for 7 days. Transcript levels of two Lhcb genes (Os03g0592500 and Os09g034650) were analyzed by RT-PCR. Ubiquitin (UBQ) was used as an internal control. C. PHYB(C364A) transgenic lines and phyB mutants exhibited a pale green phenotype under R. Visual phenotypes of WT, phyB, phyA phyB, two lines of PHYB(C364A)/aabb (#27-6- and #71-2-), and two lines of PHYB/aabb (#7-1- and #11-3-) seedlings grown under R for 7 days. Bar = 10 mm. D and E. Visual phenotypes of WT, phyB, phyA phyB, and PHYB(C364A)/Aabb (#27-6-) seedlings grown under FR (D) or R (E) for 8 days. Mutated and wild-type PHYA alleles are indicated by a and A, respectively. White arrow heads in (D) and (E) indicate the apices of coleoptiles of 8-day old seedlings. Bar = 10 mm. F. R-induced expression of Lhcb genes in the PHYB(C364A)/aabb seedlings. Transcript levels of two Lhcb genes (Os03g0592500 and Os09g034650) were analyzed by RT-PCR in the PHYB(C364A)/aabb seedlings (#27-6- #69-2-, and #71-1-). Ubiquitin (UBQ) was used as an internal control. (TIF) [file pone.0097264.s006.tif]

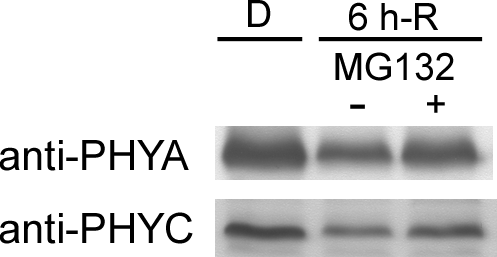

Supplement: Figure S7 — Treatment with MG132 delays light-induced degradation of phyA and phyC in rice seedlings. Four-day-old etiolated WT seedlings (D) were treated with 50 µM MG132 (+) or 0.5% DMSO (−) for 1.5 h and then exposed to R for 0 or 6 h before harvesting. Protein extracts (50 µg) from these seedlings were used to detect phyA and phyC with monoclonal anti-rye PHYA (mAR08) and anti-PHYC antibodies, respectively. (TIF) [file pone.0097264.s007.tif]
