# Supplementary material for: The Phytochrome B/Phytochrome C Heterodimer Is Necessary for Phytochrome C-Mediated Responses in Rice Seedlings
Source: PLoS One. 2014 May 22;9(5):e97264. doi: 10.1371/journal.pone.0097264 (PMC4031084; doi:10.1371/journal.pone.0097264)
Supplement: Supporting Information S1 — Quantification of relative phyB and phyC protein concentrations in rice seedlings. To establish the calibration curves of purified proteins, the purified proteins were separated by SDS-PAGE and stained with CBB R-250. Signal intensities of CBB-stained purified protein bands were analyzed using NIH image 1.62 software for PHYB-His and PHYC-His (Fig. S1A). The graph (A) was produced using the loaded amounts of proteins as X-axes and the signal intensities of CBB-stained PHYB-His or PHYC-His standard proteins as Y-axes. The regression lines were fitted as Y = 61.9X for PHYB-His and Y = 78X for PHYC-His. Therefore, given the loaded amounts of the standard proteins, the corresponding intensities of CBB-stained PHYB-His or PHYC-His were calculated according to the regression lines. For quantifying the relative levels of phyB and phyC proteins, protein extracts from 5-day-old etiolated seedlings and dilution series of standard proteins (PHYB-His or PHYC-His, they were diluted by 1000-fold for immunodetection) were separated by SDS-PAGE in the same gel. phyB and phyC were immunochemically detected with anti-PHYB and anti-PHYC antibodies, respectively. Signals intensities of the representative immunoblot for phyB (or phyC) and PHYB-His (or PHYC-His) were scanned and analyzed using NIH image 1.62 software (Figs. S1B and S1C). Because the signal intensities of immunoblots with different antibodies cannot be compared directly. They were converted into the intensities of CBB staining using the data from standard proteins (PHYB-His or PHYC-His). The graph (B) was plotted with the signal intensities of CBB-stained PHYB-His or PHYC-His proteins as the X-axis and signal intensities of immunoblots as the Y-axis. The regression line was fitted. Thus, the corresponding CBB-stained intensity per immunoblot signal for phyB and phyC in the protein extracts were calculated. Then, the graph (C) was plotted with the loaded amount of protein extracts as the X-axis and the calculated CBB signa [file pone.0097264.s008.pdf]

Supplemental information S1

(A) Relation between protein amounts and CBB intensities

Expressed PHYB protein (CBB-stained)

μl	intensity
2.5	126.8
5	326.6
7.5	430.3
10	660.9
15	916.4

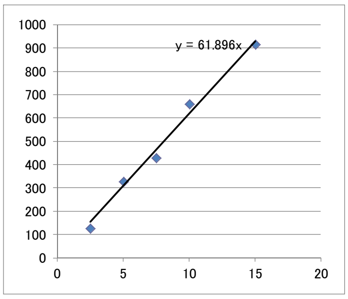

Expressed PHYC protein (CBB-stained)

μl	intensity
2.5	217.6
5	399.8
7.5	616.1
10	808.9
15	1128.8

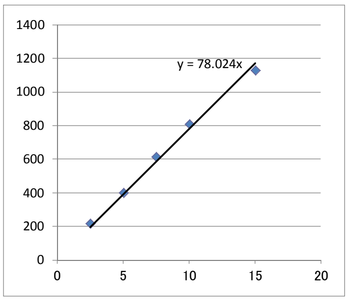

(B) Relation between intensities of CBB- and immuno-staining

PHYB (Immunostain)

Expressed PHYB (1,000 times diluted)	CBB intensity	immuno-intensity
μl	x	y
3.2	198.08	278.7
6.5	402.35	480.6
13	804.7	1007.5

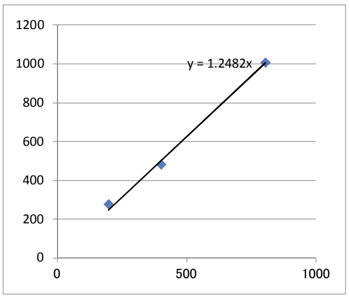

PHYC (Immunostain)

Expressed PHYC	CBB intensity	immuno-intensity
μl	x	y
3.2	249.6	235.4
6.5	507	452.5
13	1014	1036.4

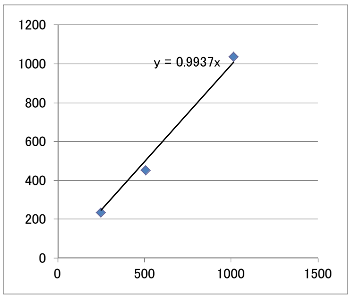

(C) Relation between protein extracts and relative amounts of PHYB or PHYC

protein ext (μg)	immuno-intensity	calculated CBB intensities
x		y
6.3	141	112.8
12.5	265	212.0
25	517	413.6
(50)	(772)	(617.6) out of the linearity

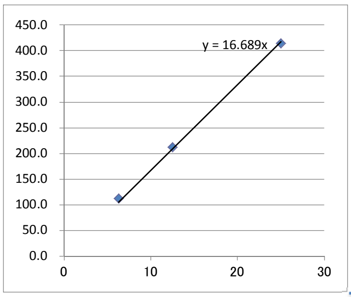

protein ext (μg)	immuno-intensity	calculated CBB intensities
x		y
6.3	63.2	63.8
12.5	178	179.8
25	324	327.3
(50)	(481)	(485.9) out of the linearity

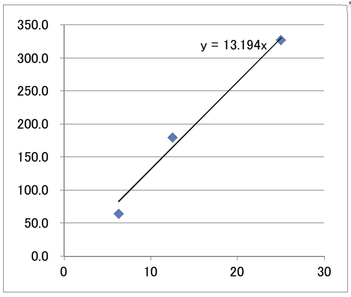

phyB/phyC
=16.7/13.2
=1.3
